# Analysis of two choir outbreaks acting in concert to characterize long- range transmission risks through SARS-CoV-2, Berlin, Germany, 2020

**DOI:** 10.1371/journal.pone.0277699

**Published:** 2022-11-17

**Authors:** Felix Reichert, Oliver Stier, Anne Hartmann, Claudia Ruscher, Annika Brinkmann, Marica Grossegesse, Markus Neumann, Dirk Werber, Marius Hausner, Mareike Kunze, Bettina Weiß, Janine Michel, Andreas Nitsche, Matthias an der Heiden, Martin Kriegel, Victor Max Corman, Terry Carleton Jones, Christian Drosten, Tobias Brommann, Udo Buchholz

**Affiliations:** 1 Department of Infectious Disease Epidemiology, Robert Koch Institute, Berlin, Germany; 2 Postgraduate Training for Applied Epidemiology (PAE), Robert Koch Institute, Berlin, Germany; 3 European Programme for Intervention Epidemiology Training (EPIET), European Centre for Disease Prevention and Control (ECDC), Stockholm, Sweden; 4 Technology, Siemens AG, Berlin, Germany; 5 Hermann Rietschel-Institute, Technical University of Berlin, Berlin, Germany; 6 State Office for Health and Social Affairs, Berlin, Germany; 7 Centre for Biological Threats and Special Pathogens, Robert Koch Institute, Berlin, Germany; 8 Local Health Authority “Berlin-Mitte”, Berlin, Germany; 9 Local Health Authority “Charlottenburg-Wilmersdorf”, Berlin, Germany; 10 Institute of Virology, Charité—Universitätsmedizin Berlin and German Centre for Infection Research (DZIF), Associated Partner Site at Charité—Universitätsmedizin Berlin, Berlin, Germany; 11 Centre for Pathogen Evolution, Department of Zoology, University of Cambridge, Cambridge, United Kingdom; 12 Choir Association of the Protestant Church Berlin-Brandenburg-schlesische Oberlausitz, Berlin, Germany; Health Directorate, LUXEMBOURG

## Abstract

**Background:**

Superspreading events are important drivers of the SARS-CoV-2 pandemic and long-range (LR) transmission is believed to play a major role. We investigated two choir outbreaks with different attack rates (AR) to analyze the contribution of LR transmission and highlight important measures for prevention.

**Methods:**

We conducted two retrospective cohort studies and obtained demographic, clinical, laboratory and contact data, performed SARS-CoV-2 serology, whole genome sequencing (WGS), calculated LR transmission probabilities, measured particle emissions of selected choir members, and calculated particle air concentrations and inhalation doses.

**Results:**

We included 65 (84%) and 42 (100%) members of choirs 1 and 2, respectively, of whom 58 (89%) and 10 (24%) became cases. WGS confirmed strain identity in both choirs. Both primary cases transmitted presymptomatically. Particle emission rate when singing was 7 times higher compared to talking. In choir 1, the median concentration of primary cases’ emitted particles in the room was estimated to be 8 times higher, exposure at least 30 minutes longer and room volume smaller than in choir 2, resulting in markedly different estimated probabilities for LR transmission (mode: 90% vs. 16%, 95% CI: 80–95% vs. 6–36%). According to a risk model, the first transmission in choir 1 occurred likely after 8 minutes of singing.

**Conclusions:**

The attack rate of the two choirs differed significantly reflecting the differences in LR transmission risks. The pooled proportion of cases due to LR transmission was substantial (81%; 55/68 cases) and was facilitated by likely highly infectious primary cases, high particle emission rates, and indoor rehearsing for an extended time. Even in large rooms, singing of an infectious person may lead to secondary infections through LR exposure within minutes. In the context of indoor gatherings without mask-wearing and waning or insufficient immunity, these results highlight the ongoing importance of non-pharmaceutical interventions wherever aerosols can accumulate.

## Introduction

While the respiratory route is considered the main transmission route of SARS-CoV-2 it can be broadly divided into short-range (SR) transmission (within 1.5 meter of the source case) and long-range (LR) transmission (any location in the room). Since the vast majority of emitted particles of an infectious person during breathing, speaking, singing, and even coughing are small enough to be able to float in the air [1, 2], and since amplifiable SARS-CoV-2 virus has been identified in aerosolized particles [3], it is thought that SR transmission is mediated by aerosol or (ballistic) droplets, whereas LR transmission is mediated by aerosols only.

LR (aerosol) transmission occurs probably exclusively indoors [4–7]. Transmission risk depends on several factors, such as the number of infectors, the viral load of the infectors, the particle emission rate and duration, the space volume and ventilation efficiency of the premises where exposure is taking place, as well as the exposed persons’ pulmonary ventilation rate and period of particle inhalation [8–10].

LR transmission has been insinuated in previous super-spreading events, such as choir rehearsals [11, 12]. However, previous outbreak investigations lacked knowledge on some of the above factors, such as particle emission measurements of involved individuals, or did not separate the contribution of SR vs. LR transmission [11–13].

In the beginning of March 2020, the first COVID-19 wave was hitting Berlin. While the first case of COVID-19 was reported in Berlin on 02 March 2020, retrospective analyses showed that, at that time, the COVID-19 epidemic was already in upward swing with a peak in infections around 15 March 2020. On Monday, 09 March 2020, the choir of a large church in Berlin (choir 1) gathered for the weekly rehearsal. The conductor led a rehearsal of another church choir (choir 2) on Thursday, 12 March 2020. On Saturday, 14 March 2020, a member of choir 1 informed the conductor of a positive test for SARS-CoV-2. The conductor informed the local health authority (LHA) about the case and the recent rehearsal. Promptly, choir members were put on 14-day quarantine, however, it turned out that many of them already had developed symptoms. Beginning on Sunday, 15 March 2020, also members of choir 2 started to feel ill.

We investigated the outbreaks with the following objectives:

- To describe the outbreaks
- To identify the source case(s)
- To examine the role of singing and speaking
- To assess the contribution of short and long-range transmission

With the analysis of the two outbreaks, we aim to provide information for the prevention of similar outbreaks in the future.

## Methods

### Epidemiological outbreak investigation

#### Study population

We considered the study population in choir 1 as all persons attending any of the last 3 rehearsals, i.e., on 09 March, 07 March, and 02 March 2020 since several transmission events might have been possible. In choir 2 the study population consisted of all persons attending the rehearsal on 12 March 2020.

#### Data collection

We conducted exploratory interviews with the conductor and key persons from the choirs. We distributed a paper-based questionnaire to the study population of choir 1 in May 2020. Questions to choir 1 included illness before the rehearsal on 09 March 2020, known possible exposure by other COVID-19 cases, travel, illness (and clinical symptoms) within two weeks before or after the rehearsal, close distance exposure to other choir members during conversations in the context of the rehearsal, seating during the rehearsal and laboratory confirmation by PCR or serology. Answers were entered pseudonymized into a database using epidata (version 2.0; https://www.epidata.dk). Data were checked for entry errors, and plausibility checks were performed before analysis.

In choir 2, structured phone interviews were conducted by one of the authors (OS), a board member of the parish in September 2020. Questions to choir 2 included illness (and clinical symptoms) within two weeks after the rehearsal on 12 March 2020, possible alternative explanations for the illness, such as travel, exposure to the conductor (distance and duration), seating during the rehearsal and laboratory confirmation by PCR or serology.

#### Identifying date of transmission and primary case (Choir 1)

To identify the date(s) of transmission in choir 1, risk ratios (RR) and population attributable fractions (PAF, ((RR-1)/RR)*proportion of cases exposed) were calculated for rehearsal dates including all laboratory-confirmed infections among all members of choir 1. To determine possible sources of transmission for choir 1 we explored all choir members who indicated an illness potentially consistent with COVID-19 starting from ten days before through two days after the identified transmission date. We assessed exposure, symptoms, laboratory-confirmation, secondary cases, and possible transmission chains.

#### Cohort study

For outbreak description and further analyses, we defined the cohorts as potentially susceptible choir members that attended the respective transmission events. Therefore, the primary cases didn´t form part of the cohort.

Additionally, choir members whose infection might have occurred somewhere else were excluded from further analyses. This applied to members of choir 1:

1. with a sequenced strain other than the outbreak strain
2. with serologically confirmed infection who had neither acute symptom onset nor positive SARS-CoV-2 PCR (if done) within two weeks after the rehearsal date.

*Case definition choir 1*. We defined a confirmed case as a person who had attended the rehearsal on 09 March 2020, was laboratory confirmed (by either PCR or serology) and had illness onset (with any of the symptoms asked), or had been tested positive by SARS-CoV-2 PCR within two weeks after the rehearsal date.

A possible case had attended the rehearsal on 09 March 2020, had acute onset of at least one respiratory or two general, non-respiratory symptoms within two weeks after the rehearsal date, but no laboratory test had been done.

*Case definition choir 2*. The same case definitions apply as for choir 1, but referring to the rehearsal on 12 March 2020, instead.

*Analysis*. Cases and non-cases are described regarding exposure, age, sex, incubation time, clinical symptoms, laboratory test results and level of care. Choir members were categorized by SR exposure (conversation with the primary case that night or seating within 1.5 m distance) or LR exposure only. We calculated overall attack rates (AR) and AR due to LR transmission only. Incubation periods and AR of the two choirs were compared by Kruskal-Wallis-Test. Analyses were performed with STATA 17.0 (Stata Corp., College Station, Texas, USA).

### Serological testing

We invited all members of choir 1 for serological testing to measure anti-SARS-CoV-2 antibodies. The outbreak of choir 2 was known 5.5 months after the rehearsal. Therefore, we invited only those members of choir 2 for serological testing, who were symptomatic after the rehearsal and did not yet have a laboratory test. Blood samples were taken 3.5 and 6.5 months after the rehearsals of choir 1 and 2, respectively.

We performed semiquantitative Euroimmun SARS-CoV-2 IgG antibody ELISA with S1 domain substrate (Euroimmun AG, Lübeck, Germany) according to manufacturer’s instructions, with the exception that we used heat-inactivated sera (56°C, 1 h) and the following cutoffs: ratio < 0.4 (negative), 0.4 ≤ ratio ≤ 3 (borderline), ratio > 3 positive. In addition, we used WANTAI SARS-CoV-2 Ab ELISA (Beijing Wantai Biological Pharmacy Enterprise, Beijing, China) for detection of complete antibodies against SARS-CoV-2 according to manufacturer’s instructions, with the exception that we applied 50 μl of serum. All samples negative by both ELISA assays, i.e., with an IgG ratio under 0.4, were considered negative. We further characterized the samples with an IgG ratio in the Euroimmun assay over 0.4 with an in-house neutralization test (NT). Details are described in A.1 in S1 Appendix. Results were considered positive when NT was positive.

For two choir 1 members, we could not obtain sera for testing and results from earlier ELISA tests performed in routine laboratories were considered.

### Sequence typing

We performed sequence typing of virus strains collected from all available specimens of PCR-confirmed cases involved in either choir outbreak as well as from cases who participated in a party, considered as a possible source outbreak of choir 1. However, most of the specimens had been discarded. We sequenced swab samples with Illumina- (Illumina, San Diego, CA, USA) and MinION (Oxford Nanopore Technologies, Oxford, United Kingdom). Sequencing methods are described in A.2 in S1 Appendix.

Phylogenetic trees were constructed by aligning the consensus sequences to chosen SARS-CoV-2 genomes sampled before April 2020 and retrieved from GISAID (https://www.gisaid.org), maximum likelihood analysis with MAFFT and visualization with Auspice, as described by the standard protocol for analysis of SARS-CoV-2 genomes provided by Nextstrain (https://www.nextstrain.org).

### Measurement of particle emission rates

We measured the particle emission of a sample of 16 participants of choir 1. Besides the presumable primary case, the conductor as well as 14 additional choir singers (3 x soprano, 5 x alto, 3 x tenor, 3 x bass) took part in the measurements. The measurements were performed in a cleanroom, which is equipped with ULPA-filters and an air change rate of more than 300 1/h, whereas the background concentration of particles (P) is 0 P/m^3^. The subjects wore special, nearly particle-free clean room compatible clothing, consisting of intermediate garments, gowns and head-covers and were placed in front of a glass pipe. At a distance of 0.81 m from the inlet of the glass pipe a particle counter (Optical Particle Counter, SOLAIR 3100, Lighthouse, CA, USA, size range 0.3->10 μm) was placed. At the other end of the glass pipe there was a filter-fan-unit with a volume flow of 400 m^3^/h. Further details regarding the test set up can be seen in a former publication Mürbe et al. [14]. Because of the distance of 0.81 m between the person and the particle counter and the time of stay of at least 0.5 s before entering the measuring probe and the particle counter, it is assumed that the particles had already reached equilibrium diameter when counted.

The subjects were asked to breath, read out a standardized text (the short story “Nordwind und Sonne”), sing two different songs (“Abschied vom Walde”, composed by Felix Mendelssohn Bartholdy and a passage from the “Liverpool Oratorio”, composed by Paul McCartney) and the tone “La” in three different volumes (piano, mezzoforte, and forte). Each task took between 10 s (singing one tone) and 30 s (other tasks) and was repeated five times. The average of the five repetitions was calculated. Four subjects were asked to perform the same tasks on two different days to investigate the intra-individual variation.

### Environmental investigation

For both choir rehearsals we collected room and ventilation data during on-site visits with the conductor and singers. Based on room measurements we calculated the room space volumes and created a room model with STAR-CCM+ (Siemens Product Lifecycle Management Software Inc.). We extracted outside temperature of the respective days (09 March 2020 for choir 1, and 12 March 2020 for choir 2) from the German Meteorological Service [15].

### Predicted Attack Rate (PAR)

Previously, three of the authors (MK, AH, UB) had published a SARS-CoV-2 adapted model by Wells and Riley [9, 16] to evaluate the risk of airborne transmission. The model is predicting AR through aerosols using four different influencing factors:

- virus related factor (viral load, particle emission rate and critical dose)
- situation-related factor (room size, time of stay, air change rate)
- susceptible person-related factor (inhalation flow rate)
- personal protection measures related factor (e.g., masks)

The model was validated with twenty-five known outbreaks [10]. For the outbreaks described here the measured particle emission rates were converted into a ratio of the emitted number of viral copies to a critical number of viral copies. With the assumption that the potentially virus laden particles are evenly distributed in the room, the infection risk via aerosol for the two choirs was calculated. The PAR model does not distinguish between SR and LR transmission but assumes equal exposure of everyone in the entire room to emitted aerosols.

### Probability of infection for short-range and long-range exposure

The infection probability depends on the exposure and differs between SR and LR exposed individuals. To our knowledge, no mathematical, physics-based model is available to relate the SR infection risk to parameters that were, or could have been, determined from the outbreak investigation. We calculated the additional SR risk originating from the primary cases by statistical inference but cannot relate it to characteristics of the setting or behavior. The LR infection risk, on the other hand, can be related mathematically to aerosol inhalation doses calculated from physical models, via an empirical dose-response function. The latter is inferred from our data in 4 steps using Wolfram Mathematica 12.2 (Wolfram Research, Inc., Champaign, Illinois, USA):

#### Step 1: Definition of exposure categories

In addition to the categorization of choir members regarding additional SR exposure, the members of choir 2 were stratified by participation in a pre-rehearsal attended by only few choir members. The subsequent main rehearsal took place with all choir members.

#### Step 2: Separation of the probabilities of infection due to short- and long-range exposure

The probability of infection for those who were exclusively LR exposed was simply the attack rate of this group. Those who had SR exposure were subject to an additional infection risk (SR risk increase) which was calculated as outlined in A.4 in S1 Appendix. The above stratification of choir 2 complicates the infection probability calculations which are described in A.5 in S1 Appendix.

#### Step 3: Particle emission rates of the primary cases and resulting inhalation doses

Based on the measured particle emission rates of the two primary cases, the cumulative particle concentration in the air was calculated assuming temporal voice activity profiles estimated from reconstruction of the course of events. The concentrations depend also on the spontaneous inactivation of virus in aerosol particles and on the ventilation efficiency during the rehearsal breaks. The number of particles inhaled by the other participants depends on the period of their attendance during the respective rehearsal and on their pulmonary ventilation rate. The calculation model is outlined in A.3 in S1 Appendix.

#### Step 4: Calculation of the number of inhaled aerosol particles (AP_50_) and virions (ID_50_) sufficient to infect 50% of exposed

The dose-response relationship between inhaled particle number and probability of infection is only modified by one parameter which can be calculated and written as the quantum *γ* (amount sufficient to infect 63.2% of exposed, AP_63_) or, alternatively, AP_50_. Both values necessarily have the same unit as the inhalation doses, i.e., the number of aerosol particles. A.6 in S1 Appendix describes the calculation of the quantum *γ* by statistical inference and its conversion to AP_50_.

Both, *γ* and AP_50_, depend on the viral load of the individual primary case. A priori, different primary cases are characterized by different dose-response curves. Only when primary cases (accidentally) have similar viral loads, they produce similar values of AP_50_. In A.7 in S1 Appendix we explain that the viral loads of our two primary cases likely differed by a factor 1 ─ 4.

The present AP_50_ estimate is specific to the outbreak strain and the viral load of the primary cases around the day of or one day before symptom onset. The estimated dependence of AP_50_ on the viral load is presented in A.7, Fig A4 in S1 Appendix. To extend the applicability of existing risk models to the variants of concern (VOC) Alpha and Delta we propose a dose adaptation estimated from the observed changes in the reproduction number *R* in A.8 in S1 Appendix.

We estimate the ID_50_ as follows: We assume the prior viral load distribution of the primary cases based on the data from Yang et al. [17]. We then estimate the infectious dose ID_50_ as the number of virions (in the sense used by Yang) inhaled by the recipients leading to infection with 50% probability (A.7 in S1 Appendix).

### Ethics statement

The study was conducted as outbreak investigation within the framework of the Prevention of Infection Act and thus exempt from submission to an ethical review committee.

## Results

### Outbreak choir 1

The choir has 96 members, including the conductor and pianist. Eighty-five choir members sent back the questionnaire (89%), 80/85 participated in serological testing, and additional two provided results from routine laboratories.

Among them, 38/85 (45%) were PCR-positive, and 64/85 (75%) were confirmed by serology yielding a total number of 65 (76%) laboratory-confirmed infections. All PCR-positive choir members with a subsequent serological test seroconverted (n = 37).

#### Identifying date of transmission and primary case (choir 1)

There were rehearsals on 02 March, 07 March and 09 March 2020. RR was highest for the 09 March rehearsal (Table 1). The PAF for the 09 March rehearsal was 91%, but reached not more than 22% for any of the other rehearsals. All but one choir members with confirmed SARS-CoV-2 infection participated in the 09 March rehearsal and 6 participated only in the 09 March rehearsal, whereas none participated only in one of the 02 or 07 March rehearsals. The one PCR- and seropositive choir member who did not participate in the 09 March rehearsal was living in the same household with another choir member who could have transmitted the virus within the household.

**Table 1:** Risk ratios of laboratory confirmed SARS-CoV-2 infection for participation in different rehearsals of choir 1, Berlin, March 2020.

	exposed			unexposed					
Rehearsal	cases n	total N	AR (%)	cases n	total N	AR (%)	RR	95% CI	p-value
09 March	64	71	90	1	14	7.1	12.6	1.91–84	<0.001
07 March, am	51	61	84	14	24	58	1.4	1.00–2.1	0.01
07 March, pm	49	60	82	14	23	61	1.3	0.95–1.9	0.05
02 March	52	69	75	12	14	86	0.9	0.68–1.1	0.40

AR = attack rate; RR = risk ratio; CI = confidence interval; am = morning; pm = afternoon

Fourteen choir members reported to have been ill within ten days before to two days after the rehearsal on 09 March 2020, rendering them potential sources for the outbreak. Of those, thirteen were either seronegative, or had no known exposure to a case outside of the choir. Furthermore, they had no travel history to a designated risk area, and none of the indicated disease episodes that began before 09 March included loss of smell or taste. Thus, it appears likely that they were not the source of this outbreak.

The 14^th^ potential source case was briefly ill on 03 and 04 March 2020, with sore throat and diarrhea. After recovery, the case attended a party in another German town approximately 580 km away on 07 March (and missed therefore the rehearsals on that day). Ten participants of that party were reported with COVID-19 and additional 30 were said to have been ill. The choir member became ill on 10 March 2020, with dry cough and high fever above 40 degrees centigrade and was later hospitalized with the need for supplemental oxygen. The son (who had not attended the party) became ill also on 10 March 2020. Sequences of specimens of members of choir 1 and from party members belonged to the same outbreak strain (with one exception; see below, “Sequence typing”).

The cases´ symptom onsets show a lognormal distribution with a peak on 12 March 2020, i.e., three days after the rehearsal, resembling a point-source outbreak (see “Outbreak description”).

**Fig 1:**
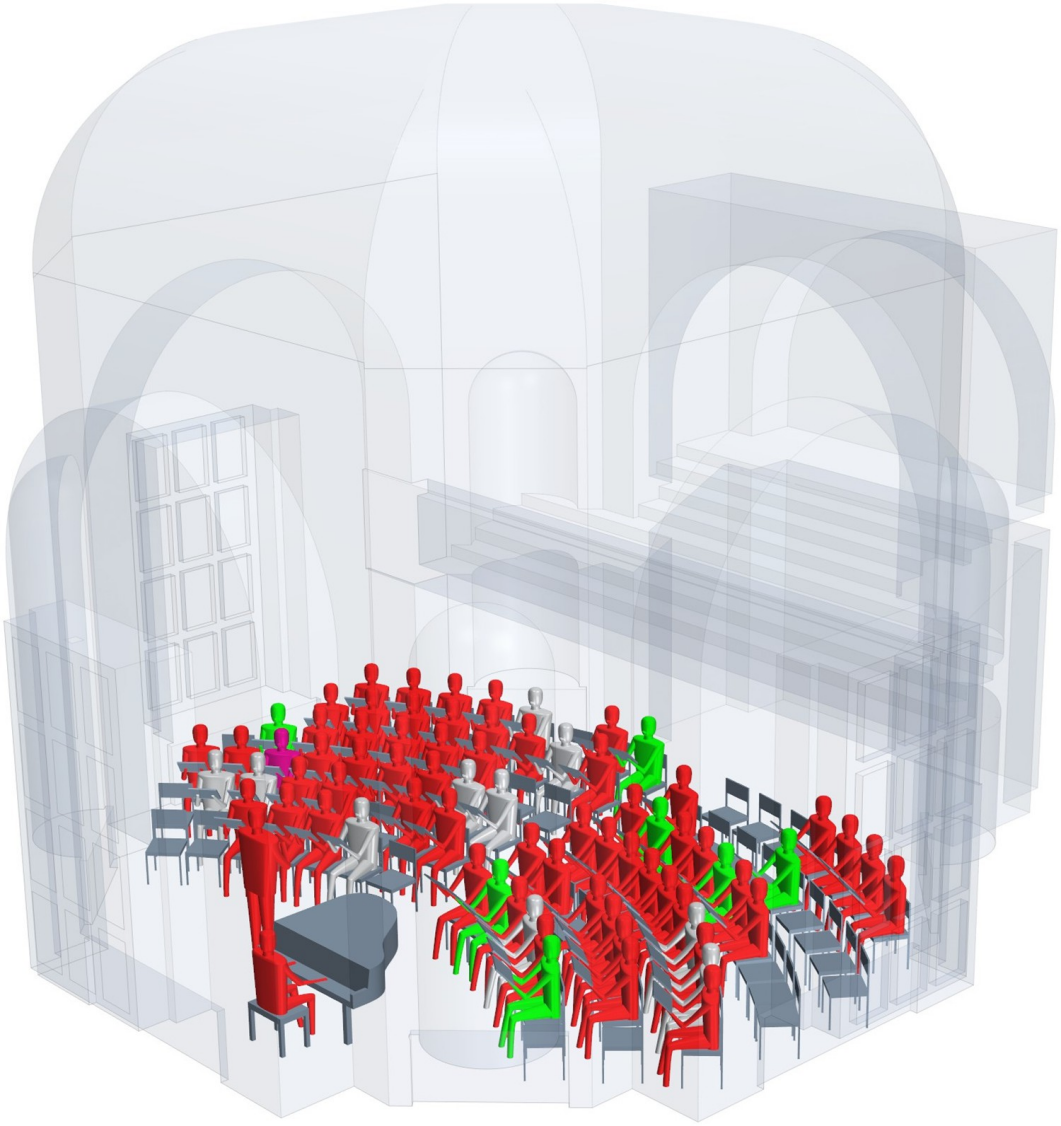
Room model of choir 1, Berlin, 09 March 2020. Presumable primary case displayed in purple, members with laboratory-confirmed SARS-COV-2-infection in red, SARS-CoV-2 seronegative members in green, members with unknown disease status in grey, standing person = conductor. Note: the seating order and distances are not identical to that of the actual rehearsal, but reconstruct the distribution of cases and non-cases in the room.

Taken together, the RR, the PAF for individual rehearsals, the evaluation of potential source cases, the distribution of symptom onsets, and the sequence results strongly suggest that choir members became infected on 09 March. The identified likely source/primary case attended a party two days before the rehearsal, likely shed virus presymptomatically, and infected inadvertently both a family member as well as the choir. The reconstructed seating order of the rehearsal on 09 March 2020 with labeling of the primary case and the infection status is shown in Fig 1.

#### Outbreak description (choir 1)

On 09 March, the choir rehearsed the “Liverpool Oratorio” for 2.5 hours with a 15-minute break when windows were tilted for ventilation. Apart from the presumed source case, 77 choir members were present, and 70 (response rate 91%) sent back the questionnaire. Five met the described exclusion criteria. Thus, 65 (84%) of 77 were included in the cohort study and further analysis. Of the 65 participants, 58 (89%) were confirmed cases, none was a possible case and 7 (11%) were non-cases (Table 2). Of the 58 confirmed cases, 34 were positive by both PCR and serology, 23 (who had no PCR test done) were confirmed by serology and one (who had no serological test done) only by PCR. Median age of the participants of the 09 March rehearsal was 51 years, 52 years among cases and 45 years among non-cases. Forty-six (71%) were female, similar among cases and non-cases. Whereas the average number of household members was the same in both groups, symptomatic secondary AR was 47% and laboratory confirmed AR was 25% among cases, but both were 0% among non-cases (Table 2).

**Table 2:** Characteristics of cases (n = 58) and non-cases (n = 7) of the 09 March rehearsal of choir 1.

	Confirmed cases n (%)	Non-cases n (%)
N	58 (89%)	7 (11%)
PCR-Test (positive/conducted)	35/45 (78%)	0/2 (0%)
Serology (positive/conducted)	57/57 (100%)	0/7 (0%)
Age (median, IQR)	52 (43–60)	45 (32–56)
Gender		
male	17 (29%)	2 (29%)
female	41 (71%)	5 (71%)
Theme / function		
soprano	19 (33%)	1 (14%)
alto	20 (34%)	4 (57%)
tenor	7 (12%)	1 (14%)
bass	10 (17%)	1 (14%)
conductor/pianist	2 (3.4%)	0
No. household members (total)	75	9
ill household members (total, attack rate)	35 (47%)	0 (0%)
lab-confirmed SARS-CoV-2 positive household members (total, attack rate)	19 (25%)	0 (0%)

IQR = Interquartile range

The most frequent symptom was fatigue (79%), followed by headache (67%) and cough (57%; see S1 Table). Dyspnea was reported by 31%. Thirteen (22%) indicated partial loss of smell and 18 (31%) indicated to have experienced complete loss of smell. Eighteen (31%) indicated partial, 14 (24%) complete loss of taste. Disease severity was mild in 39 cases (67%), moderate in 18 (31%, but only one case was diagnosed with pneumonia). One case (1.7%) was mechanically ventilated (critical). None died.

**Fig 2:**
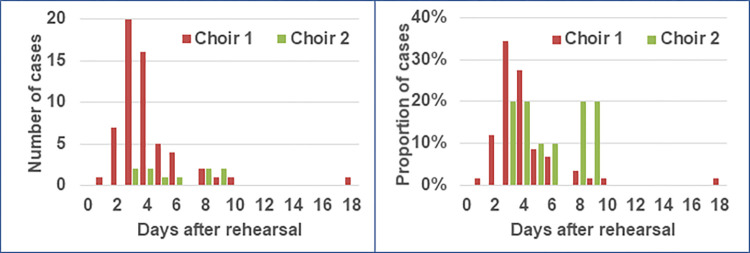
Frequency distribution of cases of choir 1 and 2 by day of symptom onset after the respective rehearsals, Berlin, March 2020. Left: frequency of number of cases, right: proportion of cases.

The mean incubation time was 4 days (range 1–18) with a median and interquartile range (IQR) of 4 days and 3–4 days, respectively (Fig 2). The case with symptom onset 18 days after the rehearsal on 09 March had a positive PCR after 11 days and disease onset after 16 days (5 days later). A household transmission cannot be excluded since the partner was singing in the choir as well and diseased on 10 March. Median duration of illness was 16 days (IQR 10–25).

Of the 65 choir members 57 had only LR exposure and 8 had LR and SR exposure. Of the 57 choir members with LR exposure only, 51 became cases (89%, 95% confidence interval (CI): 78%-96%).

### Outbreak choir 2

**Fig 3:**
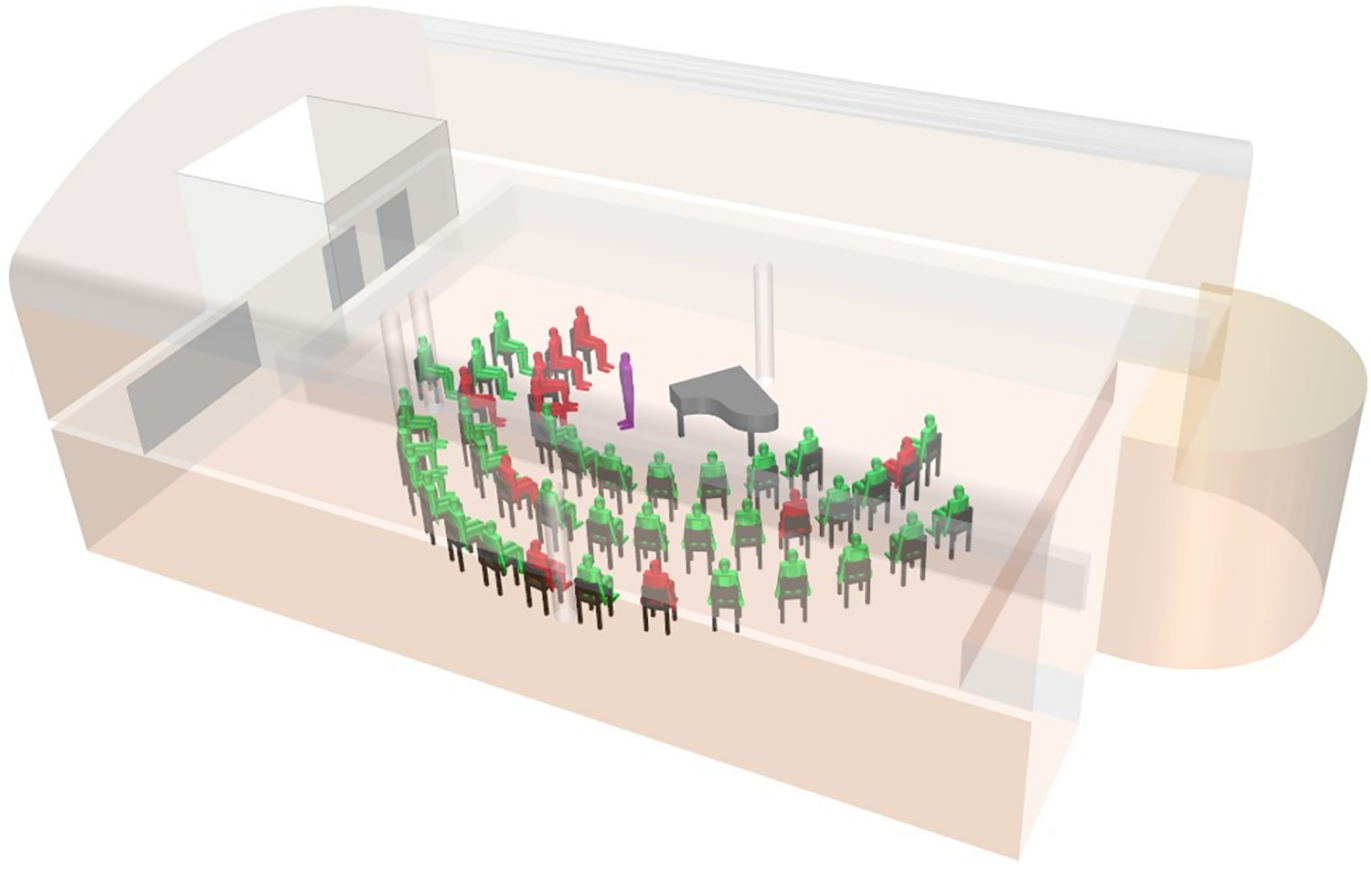
Room model of choir 2, Berlin, 12 March 2020. Presumable primary case displayed in purple, members with laboratory-confirmed SARS-CoV-2-infection in red, members with negative SARS-CoV-2 serostatus or without symptoms in green, standing person = conductor. Note: The seating order and distances are not identical to that of the actual rehearsal, but reconstruct the distribution of cases and non-cases in the room.

The conductor of choir 1 also led the rehearsal of choir 2 on 12 March 2020. He noticed first symptoms of COVID-19 after the rehearsal the same day. Apart from the conductor 42 members of the choir attended the rehearsal. The latter started with a subgroup of 13 individuals forming a chamber choir. The conductor sang the solo part of an aria or accompanied the singers at the grand piano. After 45 minutes, two windows were opened widely for a couple of minutes for ventilation during a pause and additional 29 choir members joined the group. During the following main rehearsal, the director sat mostly at the grand piano but was also leading a discussion standing near the alto singers (Fig 3).

The median age of the choir members was 56 years, the IQR was 51 to 61 years. Twenty-four (57%) of the 42 participants were female. We identified 10 confirmed cases and 32 non-cases for an AR of 24%, significantly lower than the AR of choir 1 (p<0.01). The median incubation period was 5 days (compared to 4 days in choir 1; p = 0.02; Fig 2).

Of the 42 choir members 28 had only LR exposure and 14 had LR and SR exposure. Of the 28 choir members with LR exposure only, 4 became cases.

### Sequence typing

Specimens of 18 individuals that were tested for SARS-CoV-2 after one of the outbreak events could be obtained for sequencing. Genome coverage was too low in 4 of them. For the remaining 14, sufficient numbers of reads could be generated (three participants of the party which the putative primary case of choir 1 had attended, 9 specimens of choir 1 members, one specimen of the conductor and one specimen of a choir 2 member). Except for one outlier strain, the outbreak strain was found in specimens from all party participants, 9 members of choir 1, including the conductor, and one member of choir 2 (S2 Table). Compared to reference strain Wuhan-Hu-1 (NC_045512.2) we found for all genomes of the outbreak strain 5–6 single nucleotide polymorphisms (SNPs) whereas for the outlier strain EPI_ISL_753799 only 3 SNPs at different locations (S2 Table). We identified for the outbreak strain 3, and for the outlier strain 2 synonymous amino acid substitutions (S2 Table).

**Fig 4:**
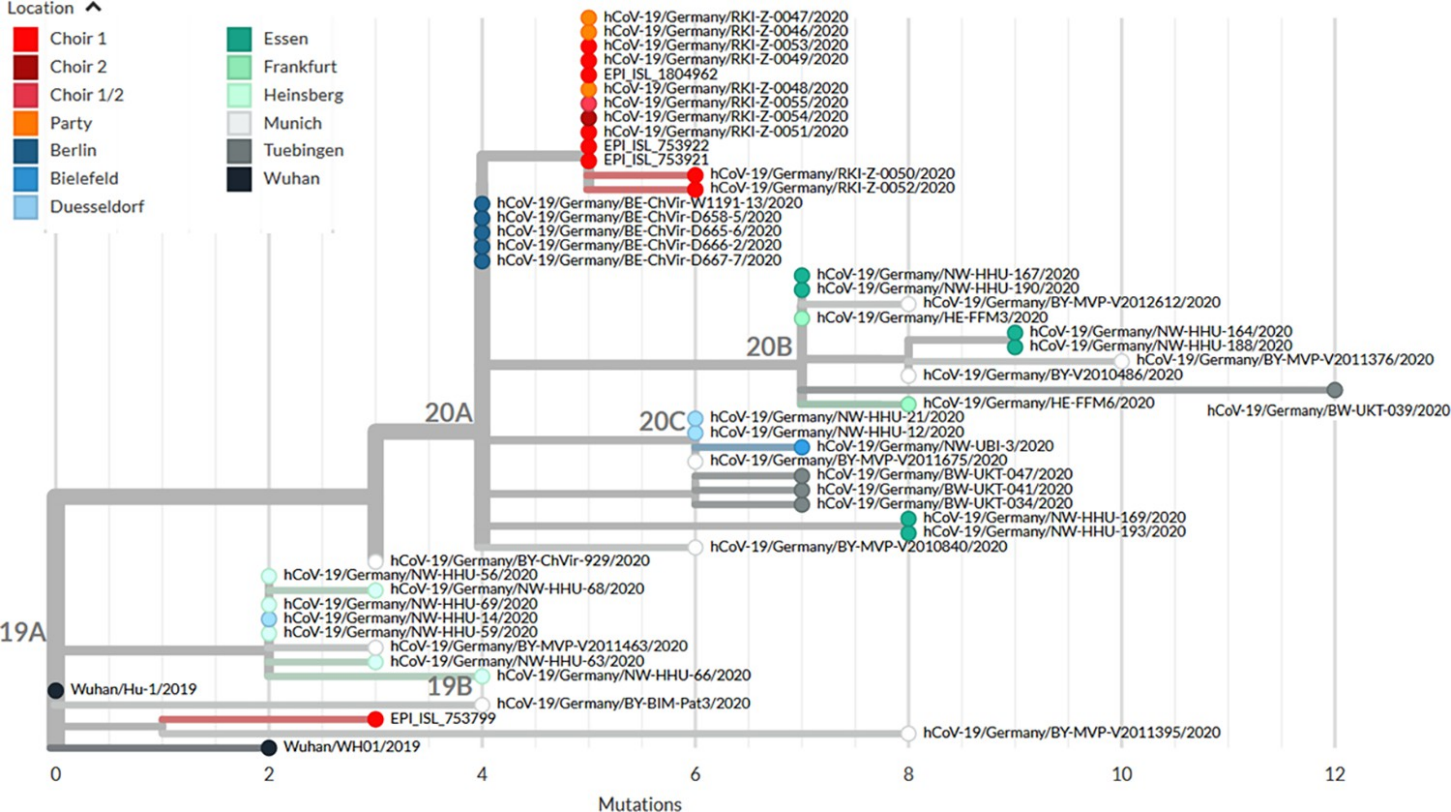
Phylogenetic relationship between the outbreak strain (choir 1, choir 2, party), the aberrant strain (choir 1), and unrelated sequences from different locations in Germany, sampled until end of March 2020. Branch length given as number of mutations.

The outlier sequence of a member of choir 1 was aberrant and even belonged to another clade. Phylogenetically, the outbreak strain clusters in GISAID clade 20A, with only one base substitution compared to genomes sampled from Berlin in March 2020. The outlier sequence with only 3 nucleotide substitutions to root strain Wuhan-Hu-1 clusters in GISAID clade 19A (Fig 4).

The patient to whom the outlier strain belonged became ill on 12 March 2020 and was tested positive for SARS-CoV-2 on 15 March 2020. However, she was no likely source of secondary cases at the choir, as her symptom onset was three days after the 09 March rehearsal, and the only secondary case was her husband who fell ill on 22 March.

### Sequence of events

**Fig 5:**
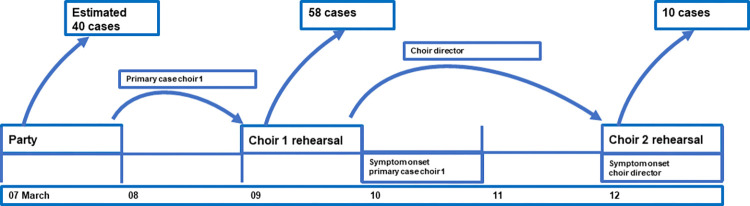
Chronology and number of cases originating from each event, Germany, March 2020.

It is most likely that the one choir member who had attended the party introduced the SARS-CoV-2 at the choir 1 rehearsal on 09 March 2020 (Fig 5). During this rehearsal the conductor must have acquired the infection and–being the sole link to choir 2—introduced the virus at the rehearsal on 12 March 2020.

### Particle emission rates

**Fig 6:**
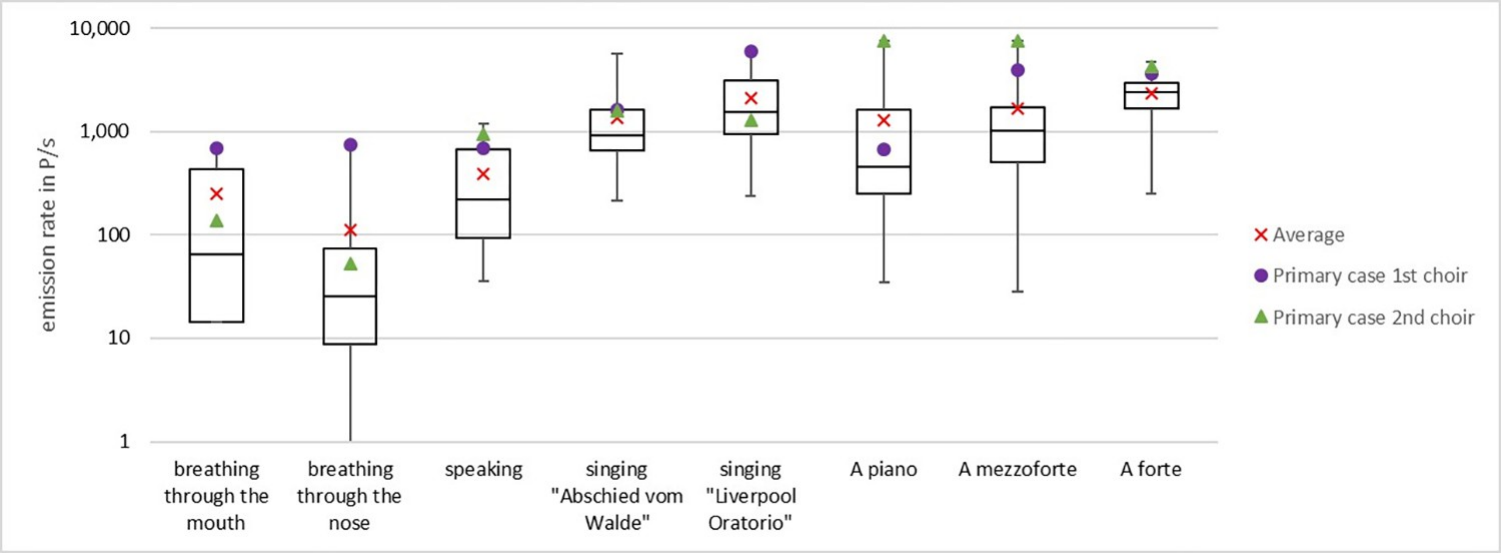
Box plots of cumulative particle emission rates for different activities among members of choir 1 (n = 16), Berlin, July- October 2020. Whiskers represent minimum/maximum values. Primary cases of choir 1 and choir 2 are indicated as points or triangles, respectively.

The measured particle emission rates were lowest for breathing through the nose, higher for speaking and reached substantially higher values when singing (Fig 6). Singing activity emitted approximately 10–30 times more particles than breathing or talking. The scatter of particle emission rates between single subjects is high. The presumable primary case of choir 1 had a particularly high particle emission rate when singing the “Liverpool Oratorio” (5979 P/s), the presumable primary case of choir 2 had a very high emission rate when singing A. The emission rate does not seem to correlate with the vocal pitch (S1 Fig). Two subjects were excluded because another particle source (e.g., beard, not wearing clothes properly, moving too much) obviously had influenced the measurement.

A repeat measurement on another day showed that the variation of emission rates was higher between individuals than between two measurements of the same individual. The presumable primary case still emitted a high number of particles when singing the “Liverpool Oratory” (2786 P/s) but was not the “top emitter” anymore (S2 Fig).

Besides the number, also the size of the particles influences the distribution of the particles in the room. More than 85% of all particles measured were smaller than 1 μm and about 99% were smaller than 3 μm. All of these are ideal airborne particles, which follow the air flow for a long time.

### Environmental investigations

The rehearsal of choir 1 took place in a room with a space volume of 1200 m^3^. A supply air volume flow of 200 m^3^/h was calculated based on the window opening during the rehearsal, the outside temperature (7°C) and the wind speed (2.5 m/s). The concentration of particles exhaled by the primary case and floating in the air was estimated to have reached 5400P/m^3^ (median) after 2.5 hours, based on the calculations described in A.3 in S1 Appendix.

The rehearsal of choir 2 took place in a room with a floor area of 234 m^2^ and a space volume of 1720 m^3^. The supply air volume was calculated from the duration of ventilation and the perceived temperature drop in the room. That night the outside temperature was 8°C, wind speed was 3.5 m/s. In the rehearsal room the concentration of the particles exhaled by the primary case and floating in the air reached 700 P/m^3^ (median) after 2.0 hours, according to our model calculation.

**Fig 7:**
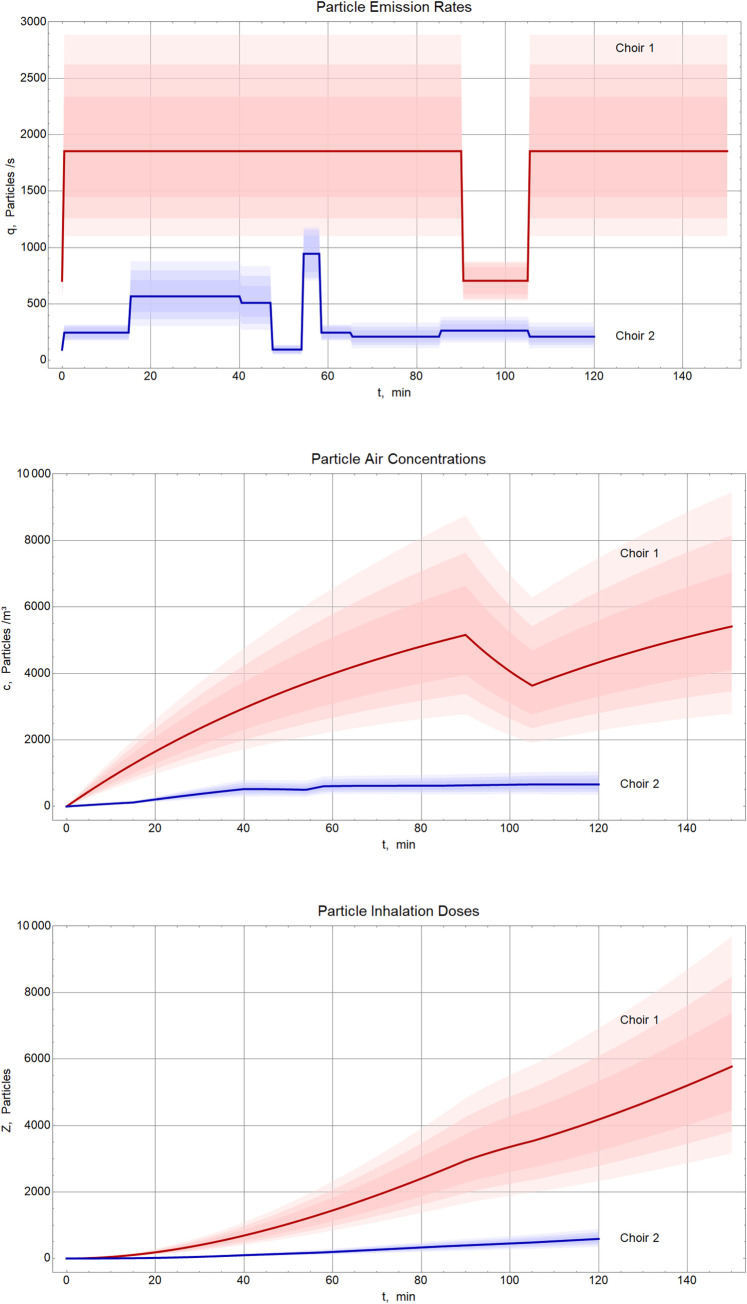
Particle emission rates of the primary cases in both choirs (top), cumulative particle concentration (emitted by the primary cases) in the room air (middle) and resulting inhalation doses (bottom) according to the model calculation described in A.3 in S1 Appendix, Berlin, March 2020. The shading indicates the uncertainty resulting from parameter estimation, showing the central credibility regions for 68.3%, 90%, and 99% coverage determined by Monte-Carlo simulations. Solid lines indicate the median values.

The large difference between the particle concentrations was primarily a consequence of the different particle emission rates of the two primary cases (approximately factor 5; Fig 7). This factor is the product of differences in vocal activity (factor 1.7) and individual particle emission rates (factor 3). Second, the rehearsal of choir 1 was half an hour longer (25%) than that of choir 2. Since inhalation doses increase with up to the square of the exposure duration (at constant emission rate), the latter difference in rehearsal duration yields a risk increase by an approximate factor 1.4. Third, the larger room of choir 2 allowed for better aerosol dilution (approximate factor 1.4). The three factors combine to an approximate ratio of 10 between the inhalation doses of choirs 1 and 2 and a consequent higher attack rate in choir 1.

### Predicted Attack Rate (PAR)

**Fig 8:**
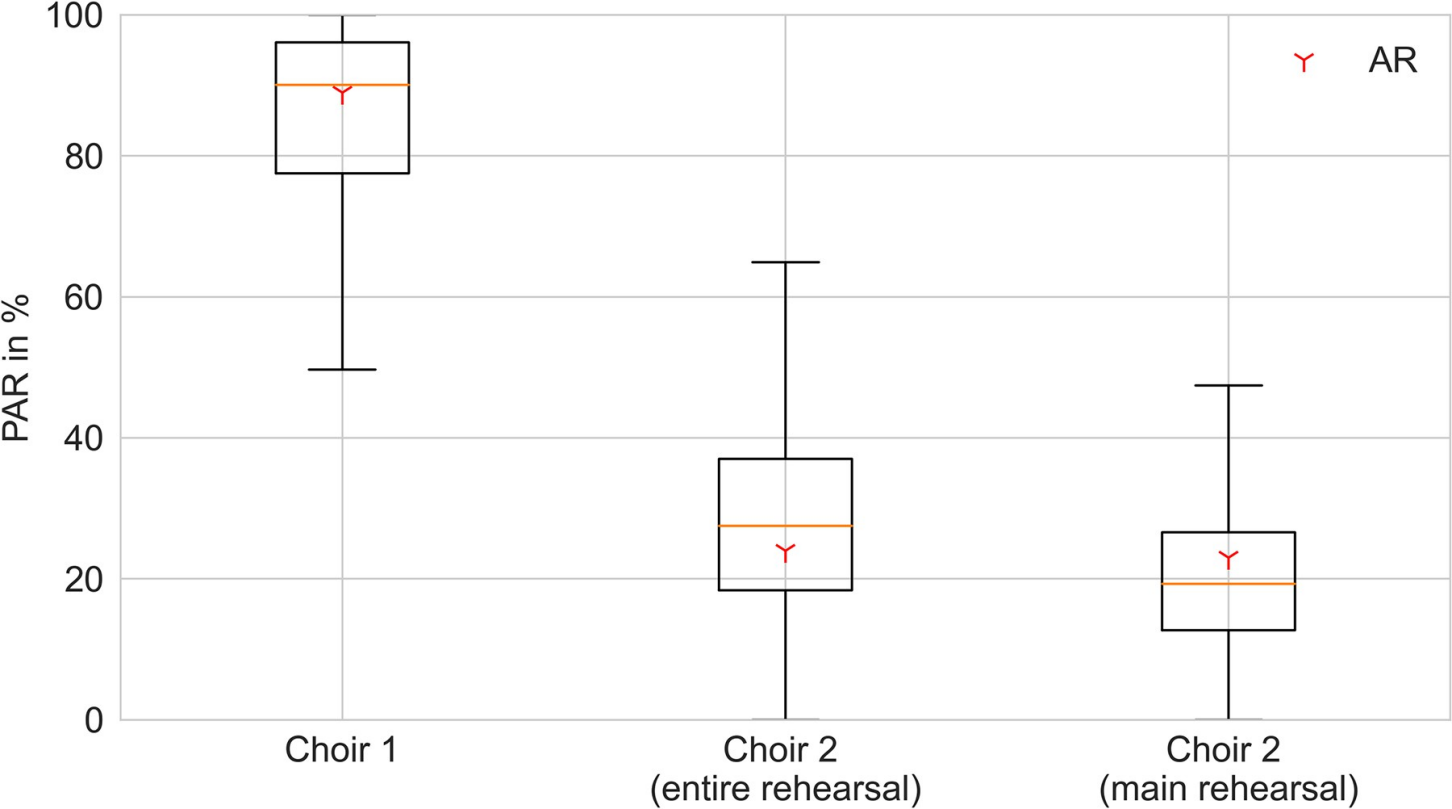
Predicted Attack Rate (PAR) for the two rehearsals from the PAR model [10], Berlin, March 2020. The error bars indicate the uncertainty resulting from parameter estimation, the red three-pointed stars indicate the observed attack rates (AR).

Some of the boundary conditions necessary to predict the attack rate with the PAR model [10] are unknown or at least uncertain (e.g. supplied air volume flow, viral load, critical dose). Therefore, a Monte-Carlo-Simulation with 100,000 runs considering the uncertainty of these boundary conditions was performed. As average values a quanta emission rate (viral emission rate per critical dose) of 1735 1/h for choir 1 and of 550 1/h for choir 2 was used. The values were adapted to the different activity levels of the primary cases of the two choirs shortly before symptom onset. The boxplots in Fig 8 show the results of this simulation. A quite good agreement between the observed attack rate (choir 1: 89%, choir 2 (entire rehearsal): 24%, choir 2 (main rehearsal only): 23%) and the calculated median results (choir 1: 90%, choir 2 (entire rehearsal): 28%, choir 2 (main rehearsal only): 19%) was found. Still, it has to be mentioned that the results cover a wide range of predicted attack rates. Unknown boundary conditions are therefore a problem if outbreaks are considered retrospectively, but it is possible to calculate a good approximation of the AR in case of a shared stay in an indoor environment around the day of symptom onset.

### Probability of infection for short-range and long-range exposure

**Fig 9:**
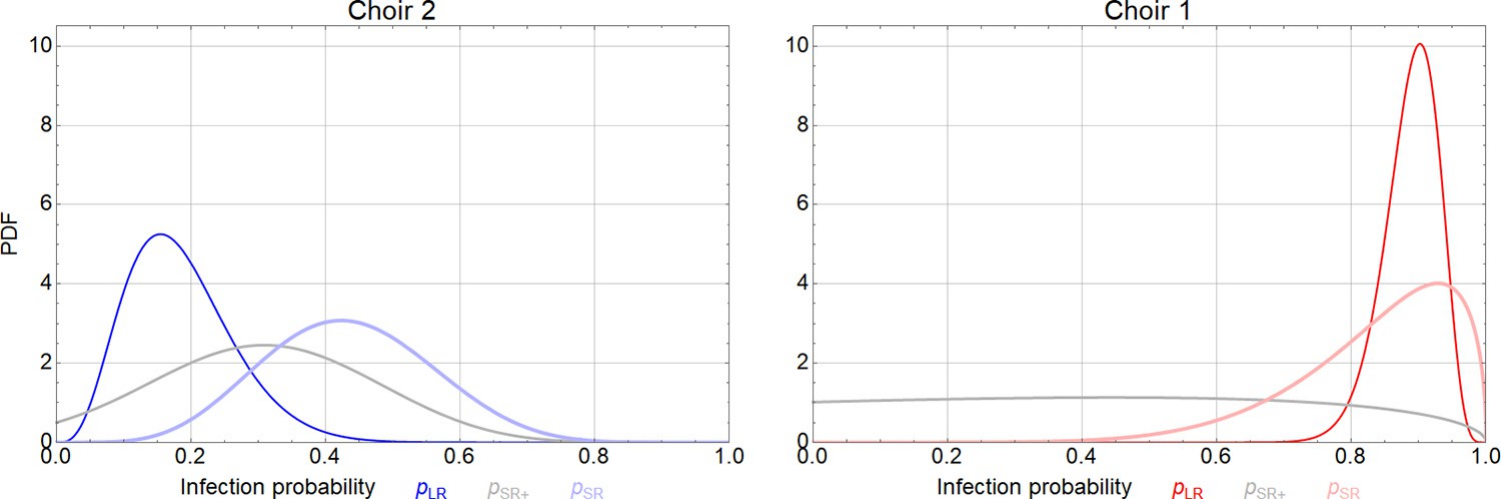
Posterior distributions of the infection risk due to long-range (LR), short-range alone (SR increase, SR+), and combined (SR) exposure in the two choir rehearsals, Berlin, March 2020.

Choir 1: Of the 58 cases, 51 had LR exposure only and 7 had additional SR exposure. Probability of infection by LR exposure was 0.90 (mode, 95% CI: 0.80 ─ 0.95) and with additional SR exposure 0.92 (mode, 95% CI: 0.55 ─ 0.99). The resulting SR risk increase is 0.44 (mode, 95% CI: 0.02 ─ 0.94). The latter estimation has a large CI because LR infection probability is 90% and the group which is additionally SR exposed is small. This prevents narrow estimation of the SR risk increase, as can be seen from the flat grey curve in Fig 9.

Choir 2: Of the 10 cases, 4 had LR exposure only whereas 6 had additional SR exposure (S3 Table). Probability of infection by LR exposure was 0.16 (mode, 95% CI: 0.06 ─ 0.36) and with additional SR exposure 0.42 (mode, 95% CI: 0.20 ─ 0.68). The resulting SR risk increase is 0.31 (mode, 95% CI: 0.04 ─ 0.62).

The posterior distributions of all probabilities, given the observed cases numbers, are shown in Fig 9. SR risk increase estimation has high uncertainty since it is backward calculated from estimates of the SR and LR infection probabilities. The large difference in LR infection probability between the two choirs reflects the one order of magnitude difference in the inhalation doses.

### Dose for infection of 50% of the exposed

**Fig 10:**
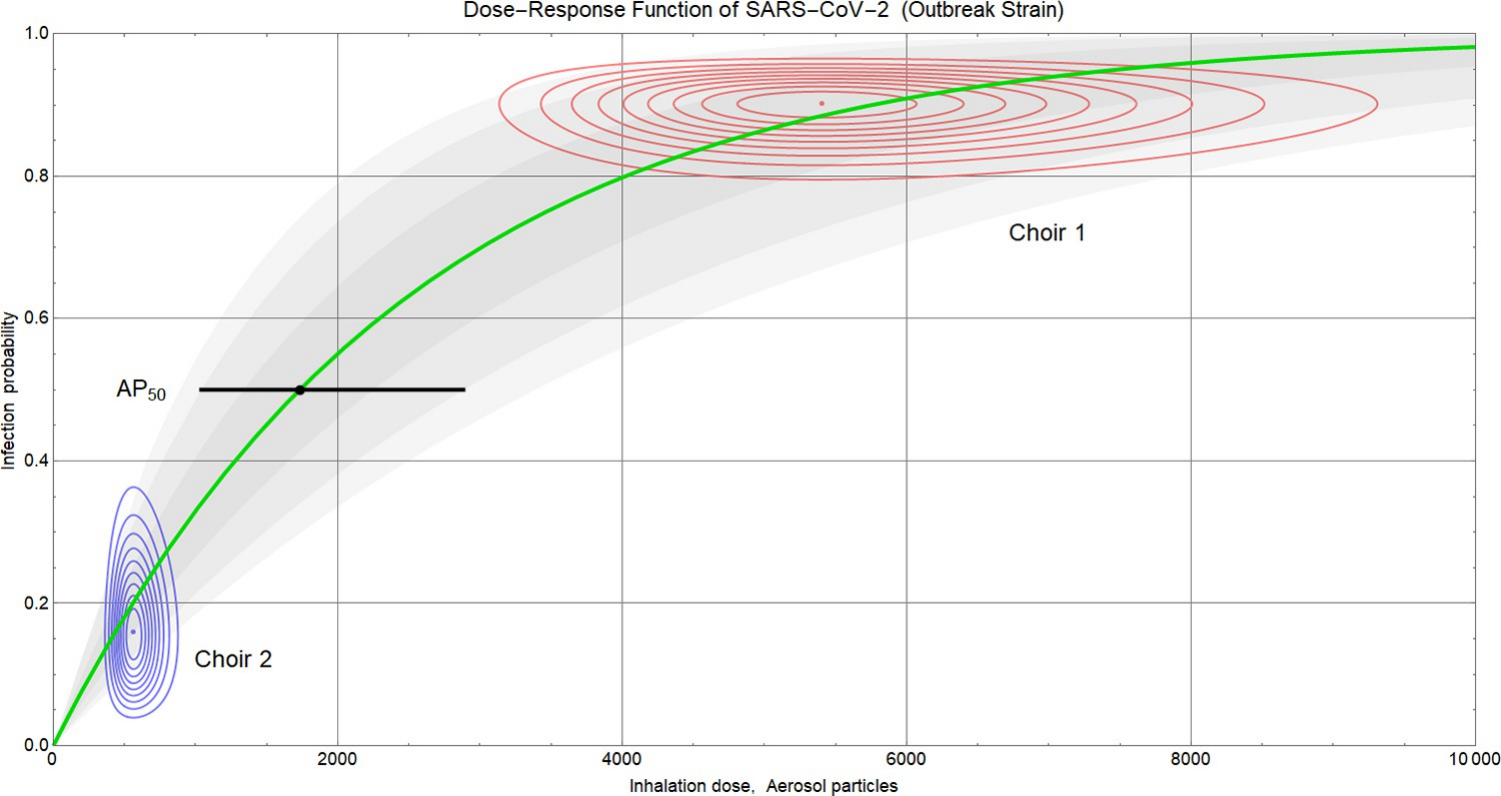
Dose-response relation Eq (8), A.6 in S1 Appendix, for different values of the quantum γ. Green curve: Median, γ = 2497 aerosol particles. Gray shaded areas: Central credibility regions for 68.3%, 95%, and 99% coverage. Black marker: Median (1731) and 95% CI (1039 ─ 2883) of the particle dose leading to infection with 50% probability (AP_50_). Red and blue contours: Linear equidistant iso-lines of the probability density of pairs (Z, p_LR_) as inferred from the two choir rehearsals.

The combination of the estimated inhalation doses (shown in Fig 7, bottom) with the estimated related infection probabilities (shown as “LR” curves in Fig 9) allows inference of the dose-response relationship, as outlined in A.6 in S1 Appendix. The result is shown in Fig 10. The red and blue dots indicate the maximum likelihood estimations from choir 1 and 2, respectively, with the surrounding lines depicting associated regions of credibility. Each dot yields a point estimation for the quantum *γ* of the exponential dose-response model. The green line represents a dose-response function closely approaching the two dots. The goodness of this fit suggests that the two independent point estimations are consistent with one and the same value of *γ* which, in turn, implies similarity of the viral loads of the primary cases. The small deviation between the dots and the curve indicates that the viral load in choir 1 was 1.2 (median) times greater than in choir 2. The median value of *γ* is 2497 (95% CI: 1499 ─ 4159) aerosol particles with equilibrium diameters (after desiccation) of 0.3 ─ 5 μm. The aerosol particle dose associated with infection of 50% of the exposed, AP_50_, is 1731 (median, 95% CI: 1039 ─ 2883; Fig 10).

Estimations of AP_50_ and the viral load are entangled. In A.6 in S1 Appendix we show that the uncertainty about viral load allows to largely uphold the latter confidence interval for the present study, and in A.7 in S1 Appendix we outline how to generalize AP_50_ estimation to risk assessment of other situations.

Knowledge of AP_50_ allows to translate particle emission rates directly to quanta emission rates [18]. Using our particle emission measurements (Fig 6) and estimation of γ (Fig 10) we obtain the following quanta emission rates (1/h) for the two primary cases: In choir 1 the median (95% CI) is 1007 (559 ─ 1796) for breathing and talking, and 4718 (2468 ─ 8784) for singing, and in choir 2 135 (63 ─ 266) for breathing, 1348 (749 ─ 2405) for talking, and 2012 (1177 ─ 3434) for singing.

Using a probabilistic approach, we find that a median of 12 virions (IQR: 6 ─ 40, 95% CI: 3 ─ 380) deposited in the respiratory tract cause infection via aerosol with 50% probability (ID_50_, A.7 in S1 Appendix).

## Discussion

We report two super-spreading events associated with choir rehearsals. Integrated epidemiological, serological, and sequencing data suggest that both outbreaks were connected and were, each, initiated by single primary cases. Both primary cases transmitted presymptomatically. The two events differed in their primary cases´ particle emission rates and duration, room space volume, and exposure time of the choir members, resulting in different LR attack rates and probabilities of infection through LR exposure.

From the epidemiological point of view, it was peculiar that the median incubation time of 4 days in choir 1 was somewhat shorter than the median incubation time of 5 days in choir 2. The incubation period of the wild type is usually reported as 5–6 days [19, 20]. Since a shorter incubation period was found to be associated with higher viral load [21], this might have contributed to the shorter incubation period in choir 1. Indeed, the data from the two outbreaks suggest that the inhalational dose exposing the choir members was substantially higher in choir 1 than in choir 2 (Fig 7). This finding also concurs with that from the Skagit choir outbreak where the median incubation period was 3 days and the attack rate of 87% was as high as in choir 1 [11].

In these two incidents, both primary cases were presymptomatic. Highest infectiousness has been shown between two days before and the day of symptom onset [22–24]. As calculated in A.7 in S1 Appendix the two primary cases likely had a viral load of at least 10^8^ copies/ml, the average peak of a person of similar age [25]. In this respect, both incidents had the right conditions for a major outbreak to occur [10]. In addition, both primary cases sang during the rehearsal, and both reached top emission rates at certain singing activities, although these may not reflect those at the day of the rehearsal. What made the difference: The particle and virus emission rates during singing were higher for the primary case in choir 1 (individual), the primary case in choir 1 did sing most of the time (behavioral), the rehearsal of choir 1 lasted 30 minutes longer and the rehearsal room of choir 1 was 30% smaller in volume than the one of choir 2 (environmental). These elements led to a factor eight difference between the estimated concentrations of exhaled small particles in the respective rooms and to a factor fourteen between the virus inhalation doses. The latter ratio is the product of the following differences: Factor 4.4 between individual virus emission rates, factor 1.7 from behavior, factor 1.4 from rehearsal durations and airing, and factor 1.4 between room sizes. In consequence, the infection probability through LR exposure was five times higher in choir 1 than in choir 2. While these setting parameters uniformly applied to all occupants of the room, we identified those choir members who had exclusive LR exposure, by our questionnaires. Thus, a total of 55 (81%) of 68 cases in the two outbreaks were due to LR transmission alone.

It may help to visualize the enormous risk of an infectious person singing when we know that singing emitted up to 30 times more particles (among all participants) compared to talking or breathing. This means that one person singing may emit the equivalent number of infectious particles as up to 30 persons in the same room talking or breathing. Our measured particle emission rates compare to Alsved et al. as well as Gregson et al. who also measured the particle emission rates during singing [26, 27]. The particle emission rates were on average 0.5 to 1.8 ng/s by Alsved el. al and 0.25 to 5.4 μg/m^3^ by Gregson et al. [26, 27]. If a breathing volume flow of 0.65 m^3^/h is assumed, the results of Gregson et al. [27] can be transformed into 0.045 to 0.98 ng/s. Furthermore, Gregson et al. [27] measured the average particle concentration to be between 0.19 P/cm^3^ to 2.0 P/cm^3^, which, with the same assumption of a breathing volume flow of 0.65 m^3^/h, can be transformed into 34 to 360 P/s, which is slightly lower than the values measured in this study, but still in the same range. In addition, the values measured by Alsved et al. [26] tend to be higher than the values measured by Gregson et al. [27] and therefore probably in between our results and the results of Gregson et al. [27]. Since the measurement set-ups are different in all studies and the breathing volume flows were not measured perfect agreement should not be expected.

It is a strength of this analysis that the two rehearsals of choir 1 and 2 build on a rare case of the same SARS-CoV-2 strain having caused two well-documented outbreaks with significantly different attack rates. Assuming similar viral loads for the two primary cases, two well-separated points of the dose-response function have been observed. This allows to calculate the dose-response relationship of LR exposure and the probability of infection with unprecedented accuracy (Fig 10).

Ultimately, one is interested in the infectious dose ID_50_. Our probabilistic estimate of 12 (median, IQR: 6 ─ 40, 95% CI: 3 ─ 380) inhaled virions agrees with a previous estimation using similar methods [28]. We estimate the number AP_50_ of inhaled particles, previously emitted by a primary case, for a 50% infection rate as 1731 (median, 95% CI: 1039 ─ 2883) aerosol particles with equilibrium diameters of 0.3 ─ 5 μm.

It has been shown that the viral load of a source case must be high for an outbreak to occur [10] and since the two primary cases were likely close to the peak of their individual infectiousness curves, the particle dose AP_50_ represents the dose under this worst case condition, leading to 50% infected among the exposed who are fully susceptible. Using it this way AP_50_ is valuable for predictive risk assessment, such as in the PAR model [10]. AP_50_ is helpful in quantifying the riskiness of environments [29] and the efficiency of safety measures for public health purposes [30]. The AP_50_ value given above is appropriate for risk calculations covering viral loads up to 10^9^ RNA copies/ml. To extend risk assessment to spreaders with greater viral loads one would better use a smaller AP_50_ value chosen according to Fig A4 in S1 Appendix. Risk calculations based on viral load and ID_50_ in units of RNA copies involve greater uncertainty than risk calculations based on particle doses and AP_50_, as outlined in A.7 in S1 Appendix.

As one example for risk assessment, we apply the PAR [10] risk calculation *a posteriori* to the rehearsal of choir 1. The PAR model predicted the observed attack rate well and estimates that—given the conditions of choir 1—a rehearsal of 8 minutes would statistically have led to at least one newly infected person among the 80 susceptible choir members. For a rehearsal of 15 min the air volume flow should have been 425 m^3^/(h*person) to prevent infections from happening, which is about 10 times the air volume flow required to keep the CO_2_-level at, or below, 1000 ppm. Volume flows like this are not achievable by ventilation through windows. Solely large concert halls with mechanical ventilation and with a significantly reduced number of visitors might offer such high-volume flows per person, as well as outdoor rehearsals.

Although we believe that, generally, CO_2_-measurements are an important tool to investigate the supplied particle-free air volume flow, our data demonstrate the limitations of CO_2_-measurements for settings like the ones described here. A person singing would produce little more CO_2_ than a person breathing or talking. However, the numbers of particles emitted will vary drastically.

We acknowledge the following limitations. First, as we measured seropositivity in choir members approximately 3.5 months and 6.5 months after the event, it is possible that we attributed some to the outbreak, which were infected later and through a different transmission event. However, as cases diseased following the rehearsal and seroprevalence in German regions did not exceed 1.2% after the first wave [31, 32], the probability that we falsely attributed cases to the outbreak is low. We may have missed few cases because the seropositivity rate declines over time. However, as even 10 months after an infection seropositivity is still maintained at 88% [33] we believe that sensitivity was still high enough after 6.5 months to capture infected choir members as cases. Lastly, as we focused on symptomatic choir members in the choir 2 outbreak we may have missed asymptomatic cases. According to a meta-analysis by Sah et al., pathogenicity of persons younger than 60 years was estimated as 70% and for persons 60 at least years old it was estimated to be 80% [34]. Thus, with 10 symptomatic cases there may be not more than two other, asymptomatic cases that we missed.

Second, although the exponential dose-response model with one parameter (*γ* or AP_50_) is the parsimonious interpretation of our data, alternative dose-response models involving tolerance or threshold doses cannot be ruled out. Inference of AP_50_ from infection probabilities involves viral load as nuisance parameter. Different combinations of particle inhalation doses and viral loads may result in identical infection probabilities or attack rates, and cannot be distinguished in a retrospective outbreak investigation.

Third, particle emission was measured when primary cases had recovered from disease. Particle emission might be substantially higher when persons are diseased [35]. This would result in a higher estimated number of (partially infectious) particles necessary to infect 50% of exposed, i.e., a greater value of AP_50_.

Fourth, calculations apply to the outbreak strain and would need adjustment for other variants. Given the higher infectivity of currently circulating VOC, adjustment results in higher infection risks and lower AP_50_. We propose an adjustment of AP_50_ for B.1.1.7 (Alpha) and B.1.617.2 (Delta) in A.8 in S1 Appendix.

Fifth, this outbreak was investigated at the beginning of the pandemic when few individuals had acquired immunity through infection and when vaccination was not yet available. In later phases of the pandemic, differences in transmission and viral dynamics for recovered or vaccinated individuals [36–38] might decrease the likelihood and proportion of infectious particles in emitters and increase the infective dose in exposed, which would influence our findings regarding the dose-response and transmission risks. However, at the time of finalizing the manuscript (August 2022) there is a considerable proportion of the population still susceptible, while in others immunity may wane and indoor mass-gathering without mask-wearing take increasingly place. In that regard, our results are still relevant.

### Conclusion

Our investigation of two choir outbreaks at the beginning of the COVID-19 pandemic has not only demonstrated the risk of LR exposure to infectious aerosols, which accumulates rapidly with increasing duration, but has disentangled the risks for long- and additional short-range exposure. Through LR exposure, an infectious person may have generated first infections among susceptible persons after only a few minutes of singing, even under room conditions large enough to accommodate a choir. For the future it is of enduring value underpinning the importance of ventilation in rooms shared by several persons to prevent transmission of SARS-CoV-2 or even other respiratory viruses as a whole.

## Supporting information

S1 Appendix Methodological details. (DOCX)

S1 Table Symptom frequency among cases of choir 1 (N = 58). (TIF)

S2 Table Mutations and amino acid substitutions in all sequenced genomes. (TIF)

S3 Table Grouping of the members of choir 2 into four cohorts, by long range (LR) and additional short range (SR+) exposure and by duration of exposure during rehearsal, Berlin, 12 March 2020. (TIF)

S1 Fig Cumulative emission while singing “Liverpool Oratorio”. (TIF)

S2 Fig Comparison of the emission rates for the subjects, whose measurements were repeated at different days. (TIF)

S1 File Description of terms. (DOCX)
