# Supplementary material for: Analysis of two choir outbreaks acting in concert to characterize long- range transmission risks through SARS-CoV-2, Berlin, Germany, 2020
Source: PLoS One. 2022 Nov 17;17(11):e0277699. doi: 10.1371/journal.pone.0277699 (PMC9671375; doi:10.1371/journal.pone.0277699)
Supplement: S1 Appendix — (DOCX) [file pone.0277699.s001.docx]

# Supporting information

# S1 Appendix. Methodological details.

## A.1 Neutralization Test

Sera were diluted in DMEM (10 % FCS, 2 mM Glutamine) in six two-fold dilutions resulting in dilutions of 1:10 up to 1:320. These dilutions were mixed 1:1 with SARS-CoV-2 (strain BetaCoV/Germany/BavPat1/2020, kindly provided by Dr. Roman Woelfel, Institute for Microbiology of the German armed forces (“Institut für Mikrobiologie der Bundeswehr”)), final virus concentration 1,000 TCID_50_ /mL and incubated at room temperature for 1 h. Subsequently, 100 µL of diluted serum-virus mix were added to wells containing 2 x 10^4^ Vero E6 cells/well (#85020206, European Collection of Authenticated Cell Cultures (ECACC), Porton Down, UK), in a 96-well plate. Each sample dilution was tested in eight replicates and cells were incubated for 5 days (37°C + 5 % CO_2_). Positive (cytopathic effect visible in light microscopy) and negative wells were counted and PRNT_50_ values calculated according to Reed and Muench [1]. A positive control consisting of a known positive sample was analyzed in parallel and back-titration of the virus stock was performed for quality control.

## A.2 Sequence typing

For Illumina Sequencing, RNA was extracted using the MagNApur 96 DNA and viral NA small volume Kit (Roche) on a MagNA Pure 96 System as recommended by the manufacturer. Sequencing was done by applying an amplicon-based sequencing approach using the 600-cycle MiSeq reagent v3 cartridge (Illumina) as described before [2]. Consensus sequence determination was done by reference by using bowtie2 version 2.3.5.1 for mapping, GATK MarkDuplicatesSpark version 4.1.4.1, for elimination of read duplicates, and by using bcftools [2].

For MinION Sequencing RNA was extracted using the QIAamp viral RNA kit (Qiagen, Hilden, Germany) in a total volume of 100 µl and transcribed to cDNA using the Superscript IV reverse transcriptase (Thermo Fisher Scientific, Darmstadt, Germany). Whole genome sequencing of the samples was performed after previous specific whole genome amplification of SARS-CoV-2 (Brinkmann et. al, manuscript in review). The libraries were prepared using the Ligation Sequencing Kit LSK-109 and loaded on Oxford Nanopore MinION SpotON Flow Cells, FLO-MIN106D, R9.4.1 (Oxford Nanopore Technologies). For transcription to FastQ sequences, computational separation of barcodes and preparation of alignments Guppy v.3.4.5 for Windows with standard parameters was used. Resulting contigs were handled with Geneious Prime 2.1 and used for calculation of accuracy and genome coverage of the consensus sequences. SNP calling was performed only on genome regions with a coverage of reads > 10 and a frequency of > 65 %.

## A.3 Calculation of particle emission, concentration and inhalation doses

The (infectious) aerosol doses inhaled by the rehearsal participants have not been measured and need to be estimated. The general approach is to estimate the emission, distribution, removal, and inhalation of particles using physical models. In this section we calculate probability distributions of the number *Z* of aerosol particles inhaled by rehearsal participants.

### **Particle emission rates**

The emission of virus-laden particles is estimated from the temporal profile of voice activity of the respective primary case. The activity levels considered are breathing (no voice activity, 0), talking (1), and singing (2). The related particle emission rates of the primary cases have been measured weeks later under controlled conditions and are summarized in Fig 6. This allows to translate the activity level *a* = 0, 1, 2 into experimentally determined particle emission rates, *q*(*τ*), at any point *τ* in time.

The temporal voice activity profile, *a*(*τ*), is estimated from reconstructions of the course of events. For choir 2, these are based on interviews and knowledge of the working style of the conductor. The rehearsals are subdivided into periods of constant activity *a*(*τ*). During most periods a mixture of activities is assumed, e.g., of 0 and 2 during singing parts. The mixing weights are estimated based on knowledge of the two choirs and the pieces sung. The mixing is treated as leading to constant, mean emission rates are calculated by linear combination of measured rates.

In summary, the temporal particle emissions of the two primary cases, *q*_1_(*τ*) and *q*_2_(*τ*), are estimated based on detailed knowledge of rehearsals 1 and 2 and experimental data shown in Fig 6. The results are shown in the top row of Fig 7.

### **Distribution and removal of particles**

The spatial distribution of aerosol particles by convection with ambient air is treated by the common assumption of well-mixed room air [8]. Neglecting potential local variations in air flow, aerosol is treated like a (more than) perfect gas instantly reaching uniform concentrations depending only on time. The underlying, implicit assumptions of infinite drift velocity, reach, and stability idealize the real properties of exhaled, airborne aerosol particles described by Chong et al. [3].

This limitation of our model could be overcome by computational fluid dynamic (CFD) simulation, which is planned to be implemented. CFD models are more realistic, including the strong dependence on initial and boundary conditions. In real life scenarios like the choir rehearsals, the convolution of uncertainties may gamble away the advantage of higher accuracy, while the expense of CFD prevents Monte Carlo simulation to account for accidental parameter variation. Therefore, we adopt, here, the seemingly simplistic well-mixed air assumption.

The air concentration of potentially infectious particles emitted by the primary cases, *c*(*τ*), is obtained through weighing up emission against dilution by non-contaminated air during the rehearsal breaks and spontaneous deactivation of virions on aerosol particles. The latter process converts initially infectious particles into harmless matter not contributing to *c*. The decay of cytopathic active virus titer on airborne particles has been found to be exponential with a half-life of *δ* = 1.15 h (median) [4].

The temporal evolution of *c* is described by

$\frac{\text{d}}{\text{d}\tau}c\left( \tau\right) = \frac{q\left( \tau\right)}{V} - \left[ L\left( \tau\right)+\frac{\text{ln} 2}{\delta} \right]c\left( \tau\right)$ (Eq 1)

with the initial condition *c*(0) = 0. *L* is the air change rate (unit: h^-1^) and V is the room space volume in m³. Time has unit h, emission rates are given in aerosol particles per hour, concentration is obtained in aerosol particles per m³ by solving Eq (1). The result is shown in the middle row of Fig 7.

### **Inhaled particle doses**

The inhalation dose is the amount of (partially infectious) aerosol particles inhaled during the rehearsal:

$Z = \int_{t_{\text{begin}}}^{t_{\text{end}}} A\left( \tau\right) c\left( \tau\right) \text{d}\tau$ (Eq 2)

where *A*(*τ*) is the pulmonary ventilation rate of the susceptible person. It is assumed as 0.65 m³/h when singing and 0.54 m³/h otherwise [5].

The rehearsal of choir 1 was attended by all participants during the entire duration, *t*_begin_ = 0 and *t*_end_ = 2.5 h. The rehearsal of choir 2 was split in two parts: The first was attended by 13 exposed persons. The second part was attended by these 13 persons and 29 further persons, 42 in total. The first group was exposed in the period from *t*_begin_ = 0 to *t*_end_ = 2.0 h and the second group from *t*_begin_ = 0.833 h to *t*_end_ = 2.0 h. This leads to different inhalation doses for cohorts 1 and 3 (attendance of entire rehearsal) versus cohorts 2 and 4 (attendance of second part only) in S3 Table. The inhalation doses for both choirs for *t*_begin_ = 0 are shown in the bottom row of Fig 7 as functions *Z*(*t*_end_). The lower doses of choir 2, cohorts 2 and 4, are not shown, they are estimated to be 26% smaller than those for cohorts 1 and 3 at *t*_end_ = 2.0 h.

The accuracy of such calculations is limited by the need to estimate parameters of Eq (1), namely the varying degree of emission, *q*(*t*), the duration and effectiveness of ventilation, *L*(*t*), and viral half-life *δ*. For those parameters, plausible ranges were determined while the actual values remain unknown. We assume *δ* to vary between 0.64 and 1.64 h [4]. The emission rate was modeled using 4 and 7 numerical parameters for choir 1 and choir 2, respectively. Air change rates *L* during the breaks were estimated from environmental considerations.

It is impossible to determine the actual inhalation doses *Z*_1,2_ during the two rehearsals, but they may be assumed being random draws from a lognormal, prior probability distribution. This assumption reflects the fact that *Z*_1,2_ is influenced by several situation-dependent parameters being statistically independent. Assuming that each one is uniformly distributed within its plausibility range leads to a lognormal distribution of *Z*. The densities of *Z*_1,2_ are calculated by Monte Carlo simulation with 100.000 random parameter sets, each, and are shown in the bottom row of Fig 7.

The possibly intuitive ansatz that *Z* is uniformly distributed within an uncertainty interval leads to a paradox since one cannot be sure the interval bounds were chosen correctly. It introduces an indeterminate error probability that the actual dose lies outside the interval, hence – in contradiction to the assumption – the probability density function (PDF) is not zero outside that interval. The more plausible lognormal distribution avoids this peculiarity and allows calculation of credibility limits for given significance levels. We obtain *Z*_1_ = 5761 aerosol particles (median, 95% CI: 3505 ─ 9470) for choir 1, *Z*_3_ = 588 (95% CI: 393 ─ 879) for choir 2, cohort 3, and *Z*_4_ = 442 (95% CI: 291 ─ 672) for cohort 4.

The particle inhalation doses depend on several factors related to the primary cases and the rehearsal settings. To separate the effects of physiological (particle emission rates) from behavioral (time shares of talking and singing) and environmental (room size, rehearsal duration, ventilation rates, virus half-life) factors we repeat the Monte Carlo simulation assuming identical emission rates for the two primary cases of 200 (2200) particles per second for breathing (talking and singing). That simulation fictitiously assumes the same individual having caused the two outbreaks, thus revealing to which extent different inhalation doses are attributable to differences in the setting, rather than physiological variation between the primary cases. The simulation results in estimated inhalation doses of $Z_{1}$ = 3871 (2680 ─ 5591) and $Z_{3}$ = 1186 (929 ─ 1514) aerosol particles with a ratio ${Z_{1}}/{Z_{3}}$ of 3.3 (mean, 95% CI: 2.1 - 5.1). Hence, we expect the environmental and behavioral differences between the settings in choir 1 and 2 to result in this factor 3.3 between the particle inhalation doses. This factor includes a factor 1.4 being the inverse space volume ratio of the rehearsal rooms.

A third Monte Carlo simulation harmonizes the environmental parameters of the two rehearsals, in addition to the physiological ones. We produce a duration of 134 min by uniform acceleration of the proceedings in choir 1 and deceleration in choir 2, assume a fictitious room space volume of 1428 m³ and air change during the break of 0.25 (357 m³). The differences remaining between the two choirs are the behavioral ones, i.e., the role of the primary case (singer vs. conductor) and the extent and temporal distribution of her vocal activity. The resulting particle inhalation doses differ by factor 1.7 (mean, 95% CI: 1.1 ─ 2.6).

Thus, the factor 3.3 accounting for the setting differences is composite of the factor 1.4 (room size), the factor 1.7 (behavior), and a remaining factor 1.4 (mean, 95% CI: 0.7 ─ 2.6) related to differences in rehearsal duration and airing. The latter factor is similar the square ratio of the rehearsal durations (1.6) which would be the expected value in case of constant emission rate at zero air change.

## A.4 Separation of short and long-range infection probabilities

The model assumption of a permanent homogenous aerosol concentration in the room air entails independence of LR aerosol inhalation doses of the position of a person in the room. The reach of aerosol (transmission) is the room size and denoted here as LR. LR exposure depends on the period of stay within contaminated air. It produces a spatially uniform, inevitable base level of infection risk monotonously increasing over time. Outdoor, LR infection risk converges to zero due to virtually infinite aerosol dilution.

Any circumstance increasing the infection risk above the LR level is denoted as short-range risk increase (SR+). The infection risk for persons exposed through SR is a composite of LR and SR+ risks. If we assign the infection probabilities *p*_SR+_ to the additional short-range and *p*_LR_ to the long-range exposure, the probability of infection for persons through SR exposure is

$p_{\text{SR}}= 1-\left( 1-p_{\mathrm{LR}} \right)\left( 1-p_{SR+} \right)$ (Eq 3).

1-*p*_SR_ is the probability of not being infected, neither by the LR base exposure, nor by the additional SR+ hazard. For persons without SR exposure *p*_SR+_ = 0, thus *p*_SR_ = *p*_LR_.

In the SR of an infectious person larger exhaled droplets may carry pathogens in amounts exceeding the aerosol load by orders of magnitude. Transmission occurs in an unpredictable, discontinuous fashion and may be more dependent on the distance than on the contact time [6]. Therefore, our SR cohort definition involves the seat position and a Boolean indicator for conversations with the primary case while neglecting the conversation length.

The derivation of the dose-response relationship in S1 Appendix A.6 involves only LR cohorts of the two choirs whose attack rates are distributed around *p*_LR_. It is possible to include SR cohorts, but not practical: Eq. (3) leads to bivariate likelihood functions of (*p*_LR_, *p*_SR+_) which are difficult to unravel, whereas *p*_SR+_ eventually is a byproduct. Therefore, the nuisance parameter *p*_SR+_ is eliminated from the estimation of *p*_LR_.

Previous outbreak investigations did not divide the susceptible population into LR and SR cohorts which impairs accurate estimation of the aerosol transmission risk [7-9].

## A.5 Infection probability distributions

The dose-response relationship of SARS-CoV-2 is calculated by Bayesian inference starting with objective (uninformative) priors [10]. In this section we calculate posterior densities, point estimates, and credibility intervals of the infection probabilities defined above.

In choir 1, out of *s*_1_ = 57 participants with only LR exposure *c*_1_ = 51 have been infected. The case number *c*_1_ depends on the unknown LR infection probability *p*_1_ according to the binomial distribution. The posterior density of *p*_1_ given the observation (*s*_1_, *c*_1_) is obtained via Bayes’ rule using the objective, Jeffreys prior

$g\left( p_{1} \right) \propto1/{\sqrt{p_{1} \left( 1-p_{1} \right)}}$ (Eq 4)

and is the beta distribution with shape parameters (1/2*+c*_1_ = 51.5, 1/2*+s*_1_*-c*_1_ = 6.5). The 2.5% and 97.5% quantiles of the beta (51.5, 6.5) distribution bound the central 95% credibility interval of *p*_1_.

As point estimate we use the median for two reasons: The probabilities of over- and underestimation are equal, and the central credibility interval contains the median for any given error probability. The median generally differs from the most likely value (mode), so that the present point estimates may differ from attack rates given in Table 1 and S3 Table. For *p*_1_ we obtain the point estimate 0.89 (median) and the 95% CI 0.80 ─ 0.95. The Bayesian credibility intervals are narrower than the Clopper-Pearson intervals given in S3 Table.

The situation in choir 2 is complicated by the existence of two LR exposure cohorts, 3 and 4, whose inhalation doses and, thus, infection probabilities are correlated. The low attack rates indicate approximate proportionality between dose and response in Eq (8). Hence, the ratio between the LR infection probabilities *p*_3_ and *p*_4_ approximately equals that of the inhalation doses *Z*_3_ and *Z*_4_.

While the Monte-Carlo simulation (S1 Appendix A.3) produced large variances for both *Z*_3_ and *Z*_4_ it yields a narrow 95% CI (0.710 ─ 0.784) for the ratio

$0.753 \approx\frac{Z_{4}}{Z_{3}} \approx\frac{p_{4}}{p_{3}} = \xi= 0.770$ (Eq 5)

so that *p*_4_ can be replaced by *ξ p*_3_. We define *p*_LR_ as being *p*_3_ and obtain the likelihood function

$l\left( p_{\text{LR}} \right) = \binom{s_{3}}{c_{3}}\binom{s_{4}}{c_{4}}{p_{\text{LR}}}^{c_{3}}\left( 1-p_{\text{LR}} \right)^{s_{3}-c_{3}}\left( \xi p_{\text{LR}} \right)^{c_{4}}\left( {1-\xi p}_{LR} \right)^{s_{4}-c_{4}}$ (Eq 6)

with *c*_3_ = 1, *s*_3_ = 8, *c*_4_ = 3, and *s*_4_ = 20. The Jeffreys prior for Eq. (6) is

$g\left( p_{\text{LR}} \right) \propto\sqrt{\frac{s_{3}\left( {1-\xi p}_{\text{LR}} \right) + \xi\left( {1-p}_{\text{LR}} \right)s_{4}}{\left( {1-p}_{\text{LR}} \right) p_{LR} \left( {1-\xi p}_{\text{LR}} \right)}}$ (Eq 7)

and is equivalent to Eq. (4) for *ξ* = 1. The posterior distribution of *p*_LR_ approximates a beta (4.38, 19.42) distribution with high accuracy (Euclidean distance 0.009 between densities). This beta distribution represents a fictitious rehearsal of 120 minutes duration with 23 participants, four of which were infected. We introduce *p*_2_ having the latter distribution as the LR infection probability for choir 2 and *Z*_2_ = *Z*_3_ as associated inhalation dose. Thus, *p*_2_ = 0.18 (95% CI: 0.06 ─ 0.36).

In analogy, the infection probabilities Eq (3) for persons exposed through SR are calculated from the observations in the SR cohorts. For choir 1, *p*_SR_= 0.86 (95% CI: 0.55 ─ 0.99) and for choir 2, *p*_SR_= 0.43 (95% CI: 0.20 ─ 0.68).

The SR risk increase *p*_SR+_ is calculated from the joint density of *p*_SR_ and *p*_LR_. For choir 1, *p*_SR+_= 0.46 (95% CI: 0.02 ─ 0.94) and for choir 2, *p*_SR+_= 0.31 (95% CI: 0.04 ─ 0.62). The densities of all probabilities are shown in Fig 9.

## A.6 Dose-response relationship

The exponential dose-response model [11] has been found to be appropriate for SARS-CoV [12] and is adopted here for SARS-CoV-2. It relates the probability *p* of infection to the inhalation dose of pathogens, *Z*, by

$p = 1-\text{e}^{-Z/\gamma}$ (Eq 8)

where *γ* is the dose causing infection with 63.2% probability and is referred to as quantum [8, 11]. The dose-response function, Eq (8), is completely known, once *γ* is known. *Z* and *γ* necessarily have the same unit, but the latter is free to choose; it could be, for instance, RNA copies, plaque-forming units, or aerosol particles. *γ* is neither a threshold dose, nor a tolerance dose, but determines how fast infection risk statistically increases at increasing inhalation dose *Z*, thus quantifying the average susceptibility of a non-immune population to infection by the respective virus strain. In this section we estimate *γ* as number of aerosol particles.

This choice has the advantage that aerosol particle number concentrations in air are largely stable over time and can be measured using commercial equipment. Although practical indoor measurements detect particles also from other sources (dust, clothing fibers, nano particles from the outside, etc.) respiratory particle concentrations and emission rates can be measured considerably easier than airborne pathogens, even under laboratory conditions. Thus, knowledge of *γ* would allow inference of disease transmission risk from readily available data. However, a given dose of particles may carry different numbers of active virus copies and, thus, entail different infection probabilities. The variability originates from particle size dispersion and statistical distribution of the viral load of different spreaders. While the size dispersion is known from measurements the viral loads of the two primary cases remain unknown. Viral load is an implicit nuisance parameter of Eq (8).

In Section A.3 we derived probability distributions of the inhalation doses *Z*_1_, *Z*_2_ and in Section A.5 distributions of the related infection probabilities *p*_1_, *p*_2_. The four distributions represent estimation uncertainty, resulting either from incomplete knowledge of parameters (*Z*) or from finite accuracy of observation (*p*_LR_). The uncertainties of *Z* and *p*_LR_ are statistically independent, despite the actual values of *Z* and *p*_LR_ being correlated by Eq (8). Therefore, the joint density of pairs (*Z*, *p*_LR_) is the product of the densities of *Z* (lognormal) and *p*_LR_ (beta distribution). The joint densities for the two rehearsals are shown as contour plots in Fig 10. From those the unknown parameter *γ* of Eq (8) is calculated by the maximum-likelihood method. Since the distribution of uncertainties around the point estimates is not bivariate normal, but a product of lognormal and beta, least-squares fitting is not applicable to the maximum-likelihood estimation of *γ*. Instead, we explicitly calculate the likelihood function of *γ* from the joint densities of observations (*Z*, *p*_LR_). The latter form the integrand in Eq (10) and the double integral is proportional to the cumulative distribution function (CDF) of *γ*.

Given a pair (*Z*, *p*_LR_), *γ* is obtained by solving Eq (8):

$\gamma^{'}\left( Z, p_{\text{LR}} \right) = -Z/{\text{ ln} \left( 1-p_{LR} \right)}$ (Eq 9).

The distribution function of *γ* is the integral of the density of (*Z*, *p*_LR_) over the region *γ’*(*Z*, *p*_LR_) ≤ γ, and derivation with respect to *γ* yields its likelihood *l*:

$l\left( \gamma\right) \propto\frac{\text{d}}{\text{d}\gamma} \int_{0}^{\infty} \int_{1-\text{e}^{-Z/\gamma}}^{1} {p_{LR}}^{c-\frac{1}{2}} \left( 1-p_{\mathrm{LR}} \right)^{s-c-\frac{1}{2}} Z^{\frac{2\mu-\ln Z}{2\sigma^{2}}-1} dp_{\mathrm{LR}}dZ$ (Eq 10).

The function *l*(γ) is similar a lognormal density, reflecting that γ is a particle dose like *Z*_1_ and *Z*_2_ just for a different AR. To obtain an analytical approximation of *l*(γ) we calculate the integral in Eq (10) for a sequence of γ values and perform a least-squares fit of the lognormal CDF before derivation with respect to *γ*. Each of the pairs (*Z*_1_, *p*_1_) and (*Z*_2_, *p*_2_) generates such a likelihood function, *l*_1_(γ) and *l*_2_(γ), for the respective rehearsal: *l*_1_(γ) ≈ PDF(lognormal(7.86, 0.31)) and *l*_2_(γ) ≈ PDF(lognormal(8.05, 0.54)). The medians of these independent estimations of γ differ by the factor 1.2 attributable to the nuisance parameter. The associated ratio between the viral loads is distributed as lognormal (0.19, 0.62) with mode 0.8, median 1.2, mean 1.5. The centering around 1 suggests that the viral loads of the primary cases accidentally were quite similar so that it makes sense to consider both rehearsals independent observations of one and the same infection quantum γ. The overall likelihood of *γ*, given the observations from both rehearsals, then is the product of the two likelihood functions:

$l\left( \gamma\right) = l_{1}\left( \gamma\right) l_{2}\left( \gamma\right)$ (Eq 11).

The function *l*(*γ*) is well approximated by a lognormal density, too, with µ = 7.823 and σ = 0.260 obtained by curve fitting. The interesting parameter of *l*(*γ*) is its median, our point estimate for *γ*. Since *l*(*γ*) is approximately lognormal the median is e*^µ^*. The posterior density of the location parameter *µ* is obtained using Bayes’ rule with an objective prior. The univariate Jeffreys prior for a lognormal distribution with known variance is flat, hence the posterior density of e*^µ^* is identical with the likelihood function of *γ* [13]. For the quantum we obtain *γ* = 2497 (median, 95% CI: 1499 ─ 4159) aerosol particles. The most likely value (mode) is 2334 aerosol particles.

The median value is 3% smaller than the quantum value previously assumed in the PIRA model [5], 2586 aerosol particles. PIRA reproduces with remarkable accuracy the attack rates of a dozen documented COVID-19 outbreaks, including the one in choir 1. Our present analysis including choir 2 corroborates the previous estimation of *γ* and adds its probability distribution and credibility range.

The second Monte Carlo simulation assuming identical particle emission rates for the two primary cases produces the pairs $\left( Z_{1},p_{1} \right)$ and $\left( Z_{2}=Z_{3},p_{2} \right)$ with the associated likelihood functions $l_{1}\left( \gamma\right)\approx$ PDF(lognormal(7.46, 0.25)) and $l_{2}\left( \gamma\right)\approx$ PDF(lognormal(8.75, 0.51)). The medians of these estimations of γ, 1738 (95% CI: 1058 ─ 2855) and 6289 (95% CI: 2303 ─ 17174), differ by the factor 3.6 attributable to the nuisance parameter. In this case the viral load must have been higher in choir 1 than in choir 2, to produce the observed LR infection probabilities, *p*_1_ and *p*_2_. Accordingly, the infection quantum $\gamma_{1}$ inferred from the outbreak in choir 1 is 30% smaller (and the viral load is 44% greater) than according to the original estimation assuming identical viral loads in both rehearsals. The CI in the latter case contains $\gamma_{1}$ with 72% probability, though. Therefore, we adhere in S1 Appendix A.7, Eq (14) to the original estimation of γ and do not further distinguish between the two primary cases. In S1 Appendix A.7, Fig. A4 we describe how to adjust γ in case greater viral loads are considered.

The aerosol particle dose leading to infection with 50% probability, AP_50_, is proportional to the quantum *γ* by the factor ln 2. Hence, the distribution of *γ* is easily transformed to the distribution of AP_50_ which is lognormal with µ = 7.456 and σ = 0.260, AP_50_ = 1731 (median, 95% CI: 1039 ─ 2883). The aerosol particles´ equilibrium diameters (after desiccation) are ≥ 0.3 µm. Such particles have diameters below 5 µm and most of them are droplet nuclei.

## A.7 Viral load of the primary cases and inference of the infectious dose

The infectious dose comprises an unknown number *ν* of virions transmitted to the susceptible individual by inhalation of *ζ* aerosol particles. We assume that the inhaled particles had the same size distribution as the particles measured in Fig 6. Let *f*(*d_p_*) be the size distribution of inhaled, or measured, particles with *d_p_* being the particle diameter and *f* the frequency. In the measurements for Fig 6 only particles larger than the threshold size *Δ* = 0.3 µm had been counted, thus all particle doses *Z* in this work refer to particles with *d_p_ ≥ Δ* (in particular, *γ* and AP_50_).

Given *f*(*d_p_*) and *Δ*, the number fraction of particles captured in counting measurements is

$\eta= \int_{\Delta}^{\infty} f\left( d_{p} \right) \text{d}d_{p}$ (Eq 12).

The number *ζ* of particles inhaled is related to the count number *Z* by

$\zeta= Z/\eta$ (Eq 13).

When particles with a diameter *d_p_* are inhaled through mouth or nose a fraction *Φ*(*d_p_*) of them will be deposited in the respiratory tract where virus transmission takes place. The average number *k* of virions contained in a particle depends on the particle size at formation and on the virion concentration *c* of the epithelial lining fluid (ELF) or saliva of the primary case.

In the period between the formation of ELF droplets [14] and their inhalation or measurement the droplets likely shrink by desiccation [15] so that the size *d_p_* measured is likely smaller than the original volume determining the virion number *k*. In our measurements 95% (by number) of the particles had diameters ≤ 1 µm.

Assuming spherical aerosol particles, *k*(*d_p_*) is calculated by

$k = \frac{4}{3}\pi\left( \frac{d_{p}}{2} \right)^{3}\varepsilon c$ (Eq 14)

where *ε* accounts for evaporative water loss and has been estimated to lie in the range 1 < *ε* ≤ 8 [16]. The total number *ν* of virions transmitted to the susceptible individual is

$\nu= Z \left\langle\Phi k \right\rangle= Z c \varepsilon\left\langle V \Phi\right\rangle= c \varepsilon Z \frac{1}{\eta} \int_{0}^{\infty} \frac{4}{3}\pi\left( \frac{d_{p}}{2} \right)^{3}\Phi\left( d_{p} \right) f\left( d_{p} \right) \text{d}d_{p}$ (Eq 15)

where angle brackets $\langle\psi\rangle$ denote the expectation value of a quantity *ψ* per *measured* particle, i.e., $\langle V\rangle$ is the average aerosol volume indicated by one particle counted. As can be seen from Eq. (15) it is necessary to estimate the particle size distribution *f*(*d_p_*) in the size range 0 < *d_p_ < Δ* not covered by the measurement.

For physical reasons, a relatively small *volume* fraction of exhaled ELF is dispersed as aerosol particles smaller than *Δ*, thus the estimation of their size distribution introduces limited uncertainty to $\left\langle V \right\rangle$. In previous studies *f*(*d_p_*) has been approximated by lognormal [17] or gamma [15] distributions. We derive a point estimate and credibility bounds for $\left\langle V \right\rangle$ by fitting gamma and lognormal distributions to the observed size bin frequencies. The fits are shown in Fig A1 for the combined exhalation of the two primary cases during the measurement of their emission rates.





Figure A1. Size distribution of aerosols exhaled by the primary cases of the two rehearsals during emission rate measurements for Fig 6. Top row: Green markers indicate cumulated particle counts with 99% error bars according to counting statistics (Poisson). Curves show fits of lognormal (left, centre) and generalized gamma (right) distribution functions. The total number n of particles is a fit parameter. Middle row: Inferred frequency distribution curves f(d_p_). Bottom row: Resulting volume fractions assuming spherical particles, and average aerosol volume per counted particle.

The left column shows a lognormal distribution with the maximum plausible mode (0.4 µm). The mode is compatible with previous studies [14, 17] but the fit does not reproduce our data well. The center column displays the best lognormal fit of our data, whose mode (0.04 µm) is unrealistic small and needs compensation by unrealistic large variance. Neither fit is convincing, but the two fits yield bounds on $\left\langle V \right\rangle$ as they produce opposite extremes of the volume fraction of particles with *d_p_ < Δ*, see bottom row of Fig A1. The most likely value of $\left\langle V \right\rangle$ is the geometric mean of the bounds, $\left\langle V \right\rangle$ ≈ 0.8 µm³.

The issues with the two lognormal fits are resolved by fitting a generalized gamma distribution to the size histogram, as shown in the right column of Fig A1. Its thin tail concentrates the main volume fraction in the physically plausible interval 0.1─10 µm where particles stay airborne. The assumed aerosol size distribution at inhalation or measurement is

$f\left( d_{p} \right) \approx236 {d_{p}}^{0.55}\text{e}^{-7.8 {d_{p}}^{0.39}}$ (Eq 16)

with a mean particle volume of 0.22 µm³ and a counting efficiency *η* = 0.28. Thus, per each particle detected 2.6 particles have been missed, so that the expected aerosol volume per counted particle is $\left\langle V \right\rangle$ ≈ 0.8 µm³, as estimated before from the lognormal fits.

The combined particle deposition efficiency in the upper, bronchial, and acinar airways is assumed as *Φ*(*d_p_*) ≡ 76%, by adding the three regional deposition efficiencies reported by Madas et al. [18]. The average deposited particle volume per counted particle is, thus, $\left\langle V\Phi\right\rangle$ = 0.6 µm³ with an uncertainty factor 2 due to the estimation of *f*(*d_p_*). Using the upper bounds for the AP_50_ particle dose, *Z* = 2883, the desiccation factor, *ε* = 8, and the average deposited particle volume, $\left\langle V\Phi\right\rangle$ = 1.2 µm³, Eq (15) yields an upper bound for the number of virions:

$\nu\leq c \left( 3\times{10}^{-8} \text{ml} \right)$ (Eq 17).

The members of choir 2 had inhaled the particle dose sufficient to infect *p*_2_ = 18% of the susceptible by LR exposure (see A.5), hence, this dose necessarily contained at least one virion in order to be potentially infectious. Since the particle dose sufficient to infect 50% of the susceptible is approximately three times (factor 50%/*p*_2_) larger, AP_50_ necessarily transmits at least *ν* ≥ 3 virions. Thus, Eq (17) yields *c* ≥ 1∙10^8^ ml^-1^ as lower bound for the viral load *c* of the primary cases.

It is emphasized that *c* is the virion concentration of exhaled bodily fluids in the moment of droplet formation, i.e., the number of RNA copies in 1 ml original ELF or saliva dispersed as aerosol. This concentration differs from viral load inferred from swab samples which also is reported as RNA copies per ml but refers to 1 ml of viral transport medium (VTM). For swab samples the virion concentrations of the respective bodily fluids or secretions differ from the reported value by the initial dilution factor resulting from immersion of the swab in VTM [19, 20].

The airborne fraction of the aerosol emitted by the primary cases has a total volume of $Z\varepsilon\left\langle V \right\rangle$ = 3.62∙10^-8^ ml in the moment of exhalation, for *Z* = AP_50_. A fraction thereof, $\Phi$ = 75.8 %, is deposited in the respiratory tract of the susceptible individual so that the reference volume $Z\varepsilon\left\langle V\Phi\right\rangle$ = 2.74∙10^-8^ ml is the proportionality constant between the viral load *c* of the primary case and the virion inhalation dose *ν* = ID_50_. Assuming a viral load of *c* = 5∙10^9^ RNA copies/ml, considered “super infectious” [21], we infer *ν* = 137. This is consistent with earlier estimations based on comparison of SARS-CoV-2 with other respiratory viruses [22] or on combination of cross-disciplinary investigation of nasopharyngeal infection with data from the Skagit Valley Chorale outbreak [23].

Viral load of the early non-VOC, wild-type lineages in saliva and ELF has been measured at the beginning of the pandemic [24, 25]. The statistical distribution of viral load in saliva of different, asymptomatic infected individuals has been reported to be lognormal with a mode (= median^3^/mean^2^) of <2 RNA copies/ml [26] which is below the qRT-PCR limit of detection. Thus, all measured viral loads are greater than the mode and lie in the distribution tail where viral loads are the less likely, the greater they are. In that investigation subjects tested positive were removed from the screening pipeline, i.e., for any positive sample there was only a single time point of viral load data. 12 % of them (approx. 170 individuals) presented viral loads *c* ≥ 1∙10^8^ ml^-1^ as shown in Fig A2 (left). Another study found that 15 % of positive respiratory samples had viral loads greater than 10^8^ ml^-1^, with occasional samples exceeding 10^10^ ml^-1^ [27]. The empirical distribution function of viral load *c* ≥ 1∙10^8^ ml^-1^, shown in Fig A2 (middle), is appropriate as prior distribution of the viral load of the two primary cases in our study which transmitted presymptomatically. Since the number *ν* of transmitted virions is proportional to the viral load *c* the posterior likelihood distribution of *ν* = ID_50_ has the same shape as the prior distribution of *c*, see Fig A2 (right): ID_50_ values are the less likely, the greater they are, see Fig A3 (left). We find that 12 (median, IQR: 6 ─ 40) virions deposited in the respiratory tract cause infection with 50% probability. Our inference agrees with a previous estimation using similar methods as ours [28]. With a coverage of 95% ID_50_ is less than 380 RNA copies. The previously assumed range of 100 ─ 1000 RNA copies for the infectious dose [15, 21, 29-32] has a likelihood of 11% according to our result which emphasizes that lower ID_50_ values are more likely than higher ones.


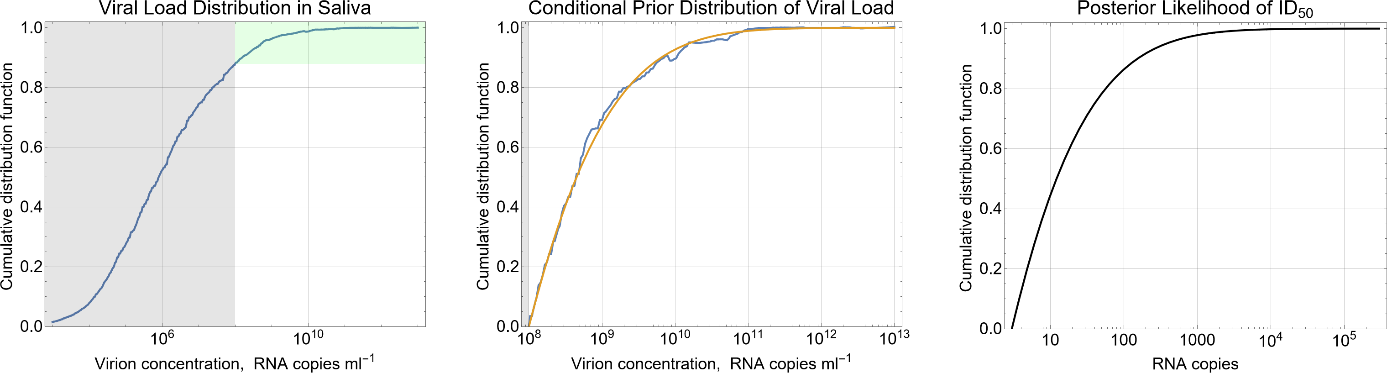


Figure A2. Left: Statistical distribution of viral load in saliva of different asymptomatic infected individuals, as reported in Fig 2B of Ref. [25]. The distribution reflects the interindividual variation at one point in time while not enfolding the temporal change of viral load in the course of infection of the same individual. This property renders the distribution apt as prior distribution of the viral load of the two primary cases in our study. Given that these had viral loads greater than 1∙10^8^ ml^-1^ the gray shaded range is irrelevant and only the range highlighted green applies. Middle: Magnification of the green range to the left, with the probability normalized to the condition c ≥ 1∙10^8^ ml^-1^. The empirical distribution function (blue) is smoothed by a fit curve (orange). Right: The number ν = ID_50_ of virions transmitted to the susceptible individual by AP_50_ particles is proportional to the viral load, given the total transmitted aerosol volume of AP_50_ as determined by our investigation. Therefore, the posterior likelihood of ID_50_ values retraces the prior distribution of the viral load values.

The dose-response model Eq (8) relates the infection probability *p* to the quanta dose

$q = -\ln\left( 1-p \right) = \frac{Z}{\gamma} = \frac{Z}{{\text{AP}_{50}}/{\ln2}} = \frac{\nu}{{\text{ID}_{50}}/{\ln2}} = Z \varepsilon\left\langle V\Phi\right\rangle\ln2\times\frac{c}{\text{ID}_{50}}$ (Eq 18)

where $\varepsilon\left\langle V\Phi\right\rangle\ln2$ = 6.6∙10^-12^ ml. Quanta doses can, thus, be objectively inferred from secondary attack rates. In S1 Appendix A.5 we derive the posterior distributions of infection probability *p* in choir 1 and 2 as *p*_1_ ~ beta (51.5, 6.5) and *p*_2_ ~ beta (4.38, 19.42), respectively. The transformed distributions of *q* yield median (95% CI) estimations of the quanta inhalation doses in the two choirs, *q*_1_ is 2.23 (1.59 ─ 3.10) and *q*_2_ is 0.19 (0.06 ─ 0.44). Quanta doses reflect the average amounts *ν* of virus carried by the inhaled aerosol particles previously exhaled by the primary case, as long as Eq (8) is assumed to be applicable. Hereby, different combinations of particle doses *Z* and viral loads *c* may produce identical *p* values, i.e., attack rates. The virus inhalation doses inferred from the secondary AR differ by a factor *q*_1_/*q*_2_ = 14 (mean, 95% CI: 4.7 ─ 38, mode: 8.6). This dose ratio is the product of the factor resulting from the different settings (mean: 3.3, including the environmental and behavioral differences, S1 Appendix A3) and another (individual) factor accounting for the different virus emission rates of the primary cases, 4.4 (mean, 95% CI: 1.3 ─ 13, mode: 2.6). The latter factor, in turn, is the product of the ratio between the individual particle emission rates and the ratio between the individual viral loads of the two primary cases. We cannot unambiguously resolve the contributions of the particle emission rate and viral load differences to the factor between the individual virus emission rates. Based on the measurements during singing the Liverpool Oratorio and breathing (Fig. 6) we assume that the particle emission rates differed by factor 3 (mean) and, hence, the viral loads differed by factor 1.5 (mean). In the second scenario considered above (S1 Appendix A3) particle emission rates were identical and viral loads differed by factor 4.4 (mean). Both variants are compatible with the prior statistical distribution of viral load.

According to our outbreak investigation the two primary cases were in the same infection stadium so that their viral loads, *c*_1_ and *c*_2_, can be assumed being independent draws from the same prior statistical distribution, such as the one shown in Fig. A2 (middle). Therefore, the ratio $x= {c_{1}}/{c_{2}}$ has a log-symmetric distribution with median 1 (i.e., the PDF of $\ln x$ is symmetric with respect to 0). The central 95% range of *x* is 0.01 ─ 100, assuming the underlying distribution of *c* shown in Fig. A2 (middle), while there is a probability of 50% that 1/3.7 ≤ *x* ≤ 3.7. With 30% probability *c*_1_ and *c*_2_ differ by a factor ≤ 2. Thus, accidental similarity of the viral loads of two primary cases in the stadium of maximal infectiousness is more likely than intuitively expected.

The estimation of quanta doses *q* using Eq (18) is generally applicable in the context of outbreak investigations, predictive risk assessment, or parametrization of epidemic models. The greatest contribution to the uncertainty of *q* is due to the ratio $c/{\text{ID}_{50}}$. The relevant variation range of *c* is 10^6^… 10^10^ ml^-1^ and the plausibility range of ID_50_ had been assumed as 10^2^… 10^3^, thus $c/{\text{ID}_{50}}$ would be uncertain within a five orders of magnitude range. This is illustrated in Fig A3 (right) by the blue curve showing the distribution of $c/{\text{ID}_{50}}$ as resulting from *c* ≥ 1∙10^6^ ml^-1^ distributed as shown in Fig A2 (left) and a lognormal prior distribution of ID_50_ around median 316 within the 95% CI 100 ─ 1000 [21]. As long as viral load needs to be assumed, forward calculations of inhalation doses in units of RNA copies [33] and infection risk predictions based on ID_50_ [34] lend themselves to probabilistic calculations [35, 36].


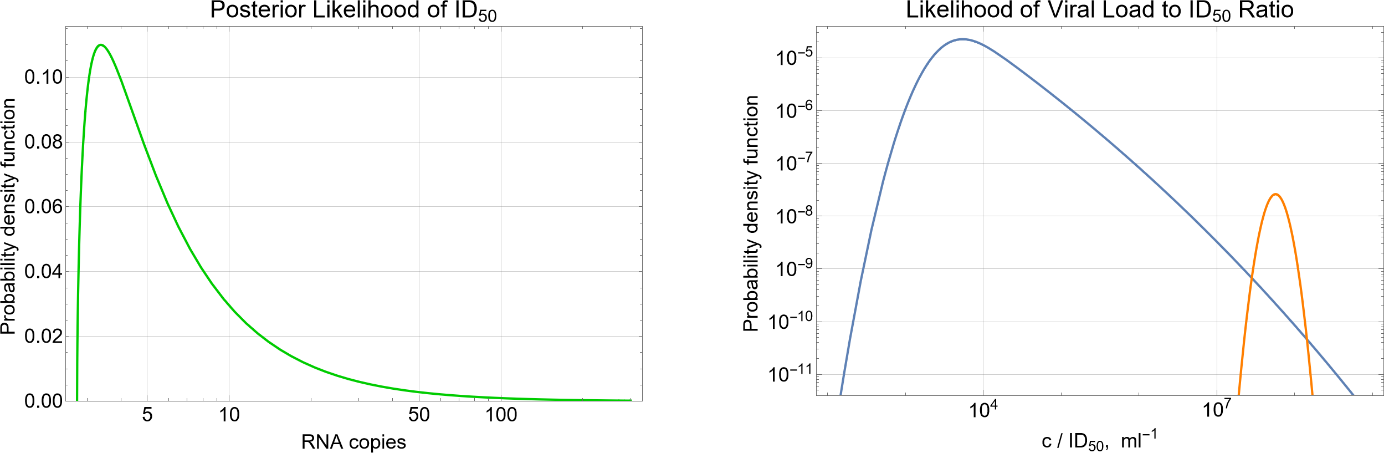


Figure A3. Left: Likelihood function of ID_50_ for respiratory inoculation as resulting from the present investigation (median: 12.2, IQR: 5.54 ─ 40.0, 95% quantile: 379 RNA copies). Right: Likelihood function of the ratio c/ID_50_ in two different scenarios. The blue curve applies to the general case where no information about the spreader is available so that her viral load potentially assumes any plausible value above the threshold for disease transmission. ID_50_ is assumed, as in prior work, to lie in the interval 100 ─ 1000 RNA copies. The width of the blue curve visualizes the absence of specific information in this scenario and indicates a need for probabilistic treatment of the resulting uncertainty. The orange curve applies to the two primary cases of our investigation for whom both viral load c and ID_50_ could be narrowed down significantly. The information from our outbreak investigation greatly reduces uncertainty as compared to the general case, we find c/ID_50_ to be 6.1∙10^7^ (median, 95% CI: 3.6∙10^7^ ─ 1.0∙10^8^) ml^-1^. This is likely the typical range in the worst case of spreaders in the stadium of maximal infectiousness and a useful input parameter for risk assessment calculations.

Eq (18) yields an alternative estimation of $c/{\text{ID}_{50}}$ directly from AP_50_ and circumventing independent prior assumptions on *c* and ID_50_:

$\frac{c}{\text{ID}_{50}} = \frac{1}{\varepsilon\left\langle V\Phi\right\rangle} \frac{1}{\text{AP}_{50}}$ (Eq 19)

With the posterior distribution of AP_50_ from S1 Appendix A.6, Eq (19) produces the orange curve in Fig A3 (right). This narrow likelihood function highlights the particular case of a spreader in the stage of maximal infectiousness. Risk assessment calculations, which typically analyze such a worst case, significantly benefit from more precise estimation of *q* when based on AP_50_ instead of *c* and ID_50_. This way, we also succeed to determine the quanta emission rates of our two primary cases with uncertainties of just a factor 3, as presented in the main paper.

Even the viral load of spreaders in the stage of maximal infectiousness shows significant interindividual variation [19]. Therefore, the AP_50_ value examined here is representative of the two primary cases investigated, but not necessarily for the whole population of potential spreaders. On the other hand, the ID_50_ likelihood function examined here is not tied to the specific viral load of our two primary cases, due to the probabilistic inference visualized in Fig A2. Therefore, solution of Eq (19) with respect to AP_50_ yields the dependence of AP_50_ on the individual viral load *c* of a spreader. The latter is calculated using the ID_50_ likelihood function from Fig A2 and is shown in Fig A4. The AP_50_ value in Fig 10 is representative of spreaders with *c* = 7.4∙10^8^ RNA copies/ml which is the 96% quantile of the statistical distribution of viral load shown in Fig A2, left. When *c* significantly exceeds 10^9^ RNA copies/ml infection probabilities are likely underestimated when using our value of AP_50_. Risk calculations targeting rare, super-infectious spreaders may, instead, use smaller values of AP_50_ which can be chosen adequately using Fig A4.


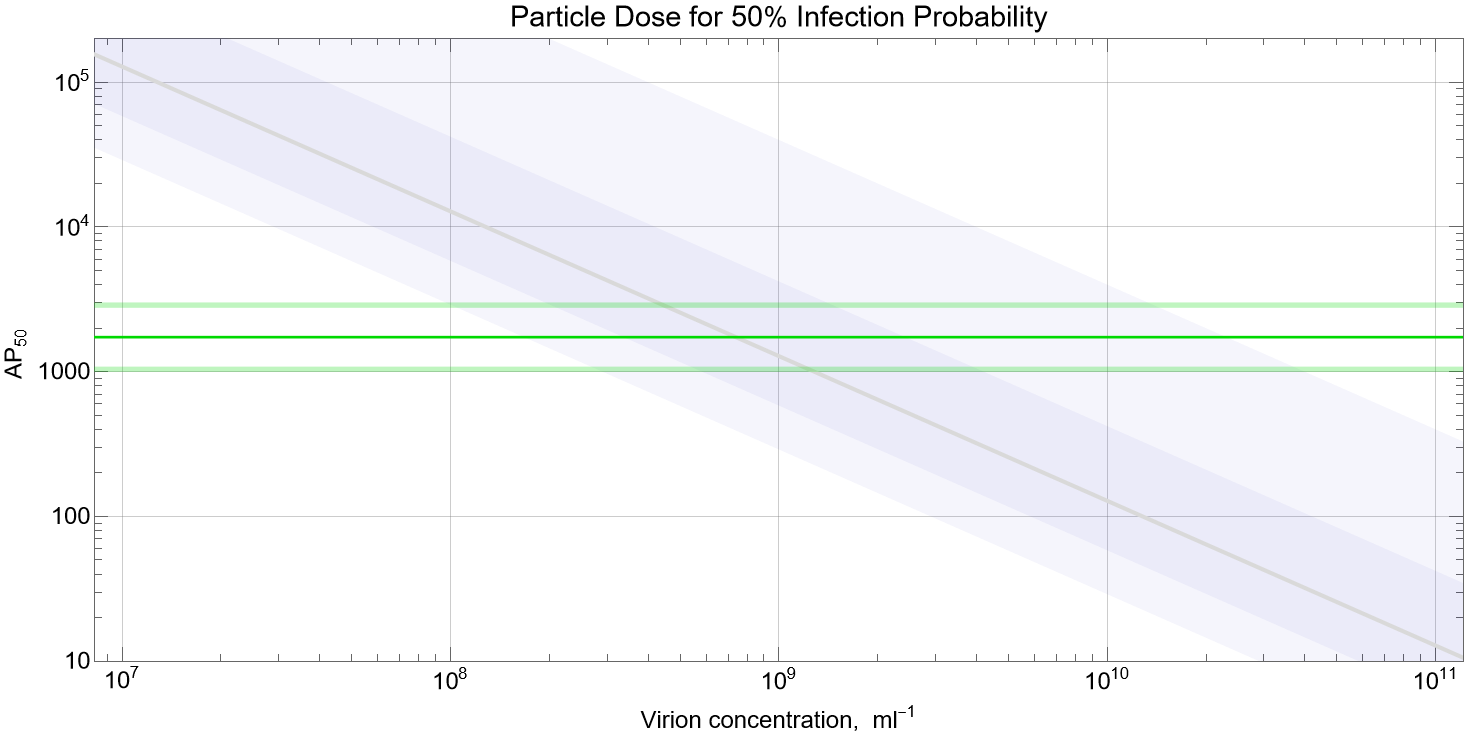


*Figure A4. Dependence of AP_50_ on the viral load of a spreader, according to Eq (19) and given the ID_50_ likelihood function from Fig. A3 (left) or Fig. A2 (right). Gray curve: Median. Gray shaded areas: Lower 95% credibility region and interquartile region. Green lines indicate the AP_50_ median and central 95% confidence interval calculated from the present outbreak and shown in Fig. 10. The intersection of the green and gray median lines is* *at a viral load of 7.4∙10^8^ RNA copies/ml.*

A peculiarity with ID_50_ is that viral load and cell culture infectivity cannot be translated directly to transmission probability [19]. The VOC Alpha and Delta produce both higher viral load and higher transmissibility than the wild type, as outlined in S1 Appendix A.8, but the increments of the two quantities are not proportional, let alone, equal. This indicates independent changes of the infectious dose ID_50_ between the variants which are difficult to predict from models.

Summarized, risk calculations based on RNA copies involve greater uncertainty than risk calculations based on particle doses and AP_50_ even if all environmental effects are treated in the same way.

## A.8 Adjustment for the Alpha and Delta variants

Present knowledge of the lineage B.1.1.7 (Alpha) allows for the possibility that the observed increase of the associated effective reproduction number *R_eff_* over the wild-type lineages is primarily a result of increased transmissibility, rather than of changes of the contact behavior or duration of infectiousness.

An additive increase in *R_eff_* appears as plausible as a multiplicative increase. The first might reflect increases in transmissibility in specific subpopulations or contexts while the latter would be expected if transmissibility had increased in all settings and individuals [37]. In this case a higher infectiousness of Alpha would likely be the cause of the increased *R_eff_* [38]. Notably, three genetic changes have been found in Alpha which are thought to result in lower infectious doses [39]. Relative to non-VOC cases, patients with the Alpha variant had viral loads that were higher by a factor of 10 and estimated cell culture infectivity that was higher by a factor of 2.6 [19].

Based on the assumption of a multiplicative increase, AP_50_ for Alpha can be estimated from AP_50_ for the wild type examined here. If both lineages, Alpha and wild-type, exhibit the same durations of infectiousness and average rates of contact between infected and susceptible individuals, the ratio between the *R_eff_* numbers is the same as the ratio between the transmission probabilities [40].

No indication has been reported that higher infection probability associated with Alpha was due to different transmission situations or behavior of exposed individuals. Since no higher mean particle intake doses should be assumed, AP_50_ for Alpha infection is expected to be smaller.

Infection probabilities in typical single transmission settings are small and, thus, approximately proportional to the doses transmitted, and inversely proportional to AP_50_. Hence, the AP_50_ values of the Alpha and wild-type lineages differ by the reciprocal of the *R_eff_* ratio.

Three independent estimations of the increase factor of *R_eff_* have been proposed [37, 38, 41]. We calculate the intersection of the three published credibility intervals from a posterior density obtained by multiplication of three associated likelihood functions, analogous to Eq (11). For each published set of a mean estimate and a 95% CI we calculate a lognormal likelihood function as a best fit to those parameters. The product of these three likelihood functions is lognormal, too, and represents the combined likelihood of the increase factor. As in the case of *γ* above, the latter function is the posterior density of the median estimate, given the results of the three studies. We obtain a multiplicative increase of *R_eff_* by 1.48 as point estimate and the central 95% CI 1.26 ─ 1.75. The four likelihood functions are displayed in Fig A5.


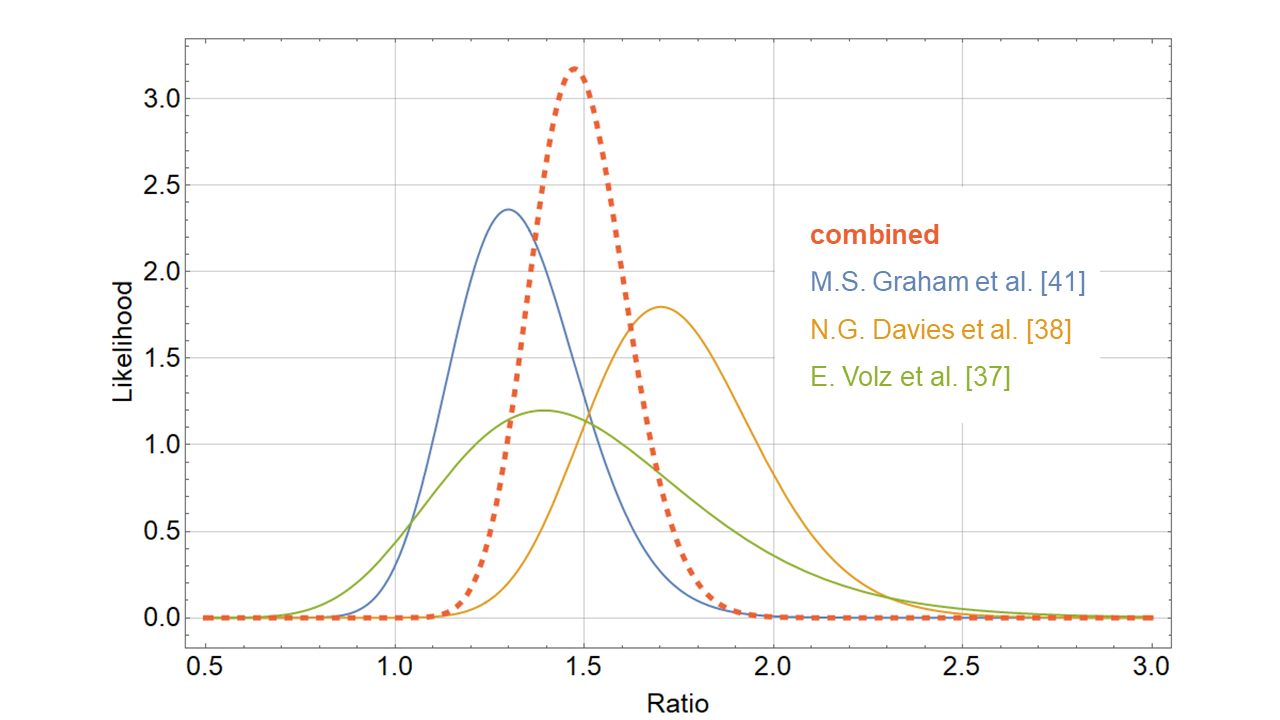


Figure A5. Multiplicative increase of R_eff_ for VOC B.1.1.7 (Alpha). Lognormal likelihood functions for the increase factor of R_eff_, modelled based on three studies (solid lines), and resulting posterior probability density (dashed line). The mode is 1.474 and the median is 1.485.

Assuming a multiplicative increase of *R_eff_* by the factor 1.48, the infectious particle dose for aerosol transmission of Alpha is estimated to be AP_50_ = 1166 (median, 95% CI: 700 ─ 1942)
aerosol particles with diameters between 0.3 and 5 µm.

A decrease of the worst-case particle dose AP_50_ may reflect a higher peak viral load, an increased infectiousness of single virions (lower ID_50_), or any combination of changes of the two factors [34, 42]. Which one contributes to which extent cannot be unraveled without additional investigation. Neither viral load, nor cell culture infectivity can be translated directly to transmission probability [19].

The lineage B.1.617.2 (Delta) has a factor 1.4 ─ 1.6 higher transmissibility than Alpha [43-47]. This factor is composite of a higher infectiousness (expressed by AP_50_) and a larger duration of infectiousness (resulting in more opportunities for secondary infections). We assume an increase of infectiousness over the Alpha variant by the factor 1.4 and apply the above consideration to derive AP_50_ = 833 (median, 95% CI: 500 ─ 1387) aerosol particles with equilibrium diameters 0.3 µm ─ 5 µm for the Delta variant. Infections with the Delta variant were found to produce higher viral load than infections with the Alpha variant [48] but this cannot be translated easily to the resulting increase of transmissibility.

References for S1 Appendix

1. Reed LJ, Muench H. A simple method of estimating fifty-percent endpoints. American Journal of Hygiene. 1938;27(3):493 ff.

2. Muller N, Kunze M, Steitz F, Saad NJ, Mühlemann B, Beheim-Schwarzbach JI, et al. Severe Acute Respiratory Syndrome Coronavirus 2 Outbreak Related to a Nightclub, Germany, 2020. Emerging infectious diseases. 2020;27(2):645-8. Epub 2020/12/03. doi: 10.3201/eid2702.204443. PubMed PMID: 33263514; PubMed Central PMCID: PMCPMC7853558.

3. Chong KL, Ng CS, Hori N, Yang R, Verzicco R, Lohse D. Extended Lifetime of Respiratory Droplets in a Turbulent Vapor Puff and Its Implications on Airborne Disease Transmission. Physical review letters. 2021;126(3):034502. Epub 2021/02/06. doi: 10.1103/PhysRevLett.126.034502. PubMed PMID: 33543958.

4. van Doremalen N, Bushmaker T, Morris DH, Holbrook MG, Gamble A, Williamson BN, et al. Aerosol and Surface Stability of SARS-CoV-2 as Compared with SARS-CoV-1. New England Journal of Medicine. 2020;382(16):1564-7. doi: 10.1056/NEJMc2004973.

5. Kriegel M, Buchholz U, Gastmeier P, Bischoff P, Abdelgawad I, Hartmann A. Predicted Infection Risk for Aerosol Transmission of SARS-CoV-2. medRxiv. 2020:2020.10.08.20209106. doi: 10.1101/2020.10.08.20209106.

6. Cortellessa G, Stabile L, Arpino F, Faleiros DE, van den Bos W, Morawska L, et al. Close proximity risk assessment for SARS-CoV-2 infection. Science of The Total Environment. 2021;794:148749. doi: <https://doi.org/10.1016/j.scitotenv.2021.148749>.

7. Charlotte N. High Rate of SARS-CoV-2 Transmission Due to Choir Practice in France at the Beginning of the COVID-19 Pandemic. Journal of voice : official journal of the Voice Foundation. 2020. Epub 2021/01/03. doi: 10.1016/j.jvoice.2020.11.029. PubMed PMID: 33386191; PubMed Central PMCID: PMCPMC7833901.

8. Hamner L, Dubbel P, Capron I, Ross A, Jordan A, Lee J, et al. High SARS-CoV-2 Attack Rate Following Exposure at a Choir Practice - Skagit County, Washington, March 2020. MMWR Morbidity and mortality weekly report. 2020;69(19):606-10. Epub 2020/05/15. doi: 10.15585/mmwr.mm6919e6. PubMed PMID: 32407303.

9. Miller SL, Nazaroff WW, Jimenez JL, Boerstra A, Buonanno G, Dancer SJ, et al. Transmission of SARS-CoV-2 by inhalation of respiratory aerosol in the Skagit Valley Chorale superspreading event. Indoor air. 2021;31(2):314-23. doi: <https://doi.org/10.1111/ina.12751>.

10. Efron B, Hastie T. Computer age statistical inference. New York, NY: Cambridge University Press; 2016.

11. Sze To GN, Chao CY. Review and comparison between the Wells-Riley and dose-response approaches to risk assessment of infectious respiratory diseases. Indoor air. 2010;20(1):2-16. Epub 2009/10/31. doi: 10.1111/j.1600-0668.2009.00621.x. PubMed PMID: 19874402; PubMed Central PMCID: PMCPMC7202094.

12. Watanabe T, Bartrand TA, Weir MH, Omura T, Haas CN. Development of a dose-response model for SARS coronavirus. Risk Anal. 2010;30(7):1129-38. Epub 05/20. doi: 10.1111/j.1539-6924.2010.01427.x. PubMed PMID: 20497390.

13. Zellner A. Bayesian and Non-Bayesian Analysis of the Log-Normal Distribution and Log-Normal Regression. Journal of the American Statistical Association. 1971;66(334):327-30. doi: 10.2307/2283931.

14. Johnson GR, Morawska L. The mechanism of breath aerosol formation. Journal of aerosol medicine and pulmonary drug delivery. 2009;22(3):229-37. Epub 2009/05/07. doi: 10.1089/jamp.2008.0720. PubMed PMID: 19415984.

15. Smith SH, Somsen GA, van Rijn C, Kooij S, van der Hoek L, Bem RA, et al. Aerosol persistence in relation to possible transmission of SARS-CoV-2. Physics of fluids (Woodbury, NY : 1994). 2020;32(10):107108. Epub 2020/11/07. doi: 10.1063/5.0027844. PubMed PMID: 33154612; PubMed Central PMCID: PMCPMC7607904.

16. Nicas M, Nazaroff WW, Hubbard A. Toward understanding the risk of secondary airborne infection: emission of respirable pathogens. Journal of occupational and environmental hygiene. 2005;2(3):143-54. Epub 2005/03/15. doi: 10.1080/15459620590918466. PubMed PMID: 15764538; PubMed Central PMCID: PMCPMC7196697.

17. Johnson GR, Morawska L, Ristovski ZD, Hargreaves M, Mengersen K, Chao CYH, et al. Modality of human expired aerosol size distributions. Journal of Aerosol Science. 2011;42(12):839-51. doi: <https://doi.org/10.1016/j.jaerosci.2011.07.009>.

18. Madas BG, Füri P, Farkas Á, Nagy A, Czitrovszky A, Balásházy I, et al. Deposition distribution of the new coronavirus (SARS-CoV-2) in the human airways upon exposure to cough-generated droplets and aerosol particles. Scientific Reports. 2020;10(1):22430. doi: 10.1038/s41598-020-79985-6.

19. Jones TC, Biele G, Mühlemann B, Veith T, Schneider J, Beheim-Schwarzbach J, et al. Estimating infectiousness throughout SARS-CoV-2 infection course. Science (New York, NY). 2021;373(6551). Epub 2021/05/27. doi: 10.1126/science.abi5273. PubMed PMID: 34035154.

20. Roque M, Proudfoot K, Mathys V, Yu S, Krieger N, Gernon T, et al. A review of nasopharyngeal swab and saliva tests for SARS-CoV-2 infection: Disease timelines, relative sensitivities, and test optimization. Journal of Surgical Oncology. 2021;124(4):465-75. doi: <https://doi.org/10.1002/jso.26561>.

21. Lelieveld J, Helleis F, Borrmann S, Cheng Y, Drewnick F, Haug G, et al. Model Calculations of Aerosol Transmission and Infection Risk of COVID-19 in Indoor Environments. International journal of environmental research and public health. 2020;17(21). Epub 2020/11/07. doi: 10.3390/ijerph17218114. PubMed PMID: 33153155; PubMed Central PMCID: PMCPMC7662582.

22. Karimzadeh S, Bhopal R, Nguyen Tien H. Review of infective dose, routes of transmission and outcome of COVID-19 caused by the SARS-COV-2: comparison with other respiratory viruses. Epidemiol Infect. 2021;149:e96. Epub 2021/04/15. doi: 10.1017/s0950268821000790. PubMed PMID: 33849679; PubMed Central PMCID: PMCPMC8082124.

23. Basu S. Computational characterization of inhaled droplet transport in the upper airway leading to SARS-CoV-2 infection. medRxiv. 2020:2020.07.27.20162362. doi: 10.1101/2020.07.27.20162362.

24. Blot M, Jacquier M, Manoha C, Piroth L, Charles PE. Alveolar SARS-CoV-2 viral load is tightly correlated with severity in COVID-19 ARDS. Clinical infectious diseases : an official publication of the Infectious Diseases Society of America. 2020. Epub 2020/08/10. doi: 10.1093/cid/ciaa1172. PubMed PMID: 32770223; PubMed Central PMCID: PMCPMC7454337.

25. Yang Q, Saldi TK, Gonzales PK, Lasda E, Decker CJ, Tat KL, et al. Just 2% of SARS-CoV-2&#x2212;positive individuals carry 90% of the virus circulating in communities. Proceedings of the National Academy of Sciences. 2021;118(21):e2104547118. doi: doi:10.1073/pnas.2104547118.

26. Correction for Yang et al., Just 2% of SARS-CoV-2&#x2212;positive individuals carry 90% of the virus circulating in communities. Proceedings of the National Academy of Sciences. 2022;119(7):e2123482119. doi: doi:10.1073/pnas.2123482119.

27. Kleiboeker S, Cowden S, Grantham J, Nutt J, Tyler A, Berg A, et al. SARS-CoV-2 viral load assessment in respiratory samples. Journal of clinical virology : the official publication of the Pan American Society for Clinical Virology. 2020;129:104439. Epub 2020/07/17. doi: 10.1016/j.jcv.2020.104439. PubMed PMID: 32674034; PubMed Central PMCID: PMCPMC7235577.

28. Bazant MZ, Bush JWM. A guideline to limit indoor airborne transmission of COVID-19. Proceedings of the National Academy of Sciences. 2021;118(17):e2018995118. doi: doi:10.1073/pnas.2018995118.

29. Kriegel M, Hartmann A, Buchholz U, Seifried J, Baumgarte S, Gastmeier P. SARS-CoV-2 Aerosol Transmission Indoors: A Closer Look at Viral Load, Infectivity, the Effectiveness of Preventive Measures and a Simple Approach for Practical Recommendations. International journal of environmental research and public health. 2021;19(1). Epub 2022/01/12. doi: 10.3390/ijerph19010220. PubMed PMID: 35010484; PubMed Central PMCID: PMCPMC8750733.

30. Gkantonas S, Zabotti D, Mesquita L, Mastorakos E, M. de Oliveira P. airborne.cam: a risk calculator of SARS-CoV-2 aerosol transmission under well-mixed ventilation conditions2021.

31. Riediker M, Monn C. Simulation of SARS-CoV-2 Aerosol Emissions in the Infected Population and Resulting Airborne Exposures in Different Indoor Scenarios. Aerosol and Air Quality Research. 2021;21(2):200531. doi: 10.4209/aaqr.2020.08.0531.

32. Schijven J, Vermeulen LC, Swart A, Meijer A, Duizer E, Husman AMdR. Quantitative Microbial Risk Assessment for Airborne Transmission of SARS-CoV-2 via Breathing, Speaking, Singing, Coughing, and Sneezing. Environmental Health Perspectives. 2021;129(4):047002. doi: doi:10.1289/EHP7886.

33. Buonanno G, Robotto A, Brizio E, Morawska L, Civra A, Corino F, et al. Link between SARS-CoV-2 emissions and airborne concentrations: Closing the gap in understanding. Journal of Hazardous Materials. 2022;428:128279. doi: <https://doi.org/10.1016/j.jhazmat.2022.128279>.

34. Riediker M, Briceno-Ayala L, Ichihara G, Albani D, Poffet D, Tsai DH, et al. Higher viral load and infectivity increase risk of aerosol transmission for Delta and Omicron variants of SARS-CoV-2. Swiss medical weekly. 2022;152:w30133. Epub 2022/01/13. doi: 10.4414/smw.2022.w30133. PubMed PMID: 35019196.

35. Buonanno G, Morawska L, Stabile L. Quantitative assessment of the risk of airborne transmission of SARS-CoV-2 infection: Prospective and retrospective applications. Environ Int. 2020;145:106112. doi: 10.1016/j.envint.2020.106112. PubMed PMID: 32927282; PubMed Central PMCID: PMCPMC7474922.

36. Mikszewski A, Stabile L, Buonanno G, Morawska L. Increased close proximity airborne transmission of the SARS-CoV-2 Delta variant. Science of The Total Environment. 2022;816:151499. doi: <https://doi.org/10.1016/j.scitotenv.2021.151499>.

37. Volz E, Mishra S, Chand M, Barrett JC, Johnson R, Geidelberg L, et al. Transmission of SARS-CoV-2 Lineage B.1.1.7 in England: Insights from linking epidemiological and genetic data. medRxiv. 2021:2020.12.30.20249034. doi: 10.1101/2020.12.30.20249034.

38. Davies NG, Abbott S, Barnard RC, Jarvis CI, Kucharski AJ, Munday JD, et al. Estimated transmissibility and impact of SARS-CoV-2 lineage B.1.1.7 in England. Science (New York, NY). 2021;372(6538):eabg3055. doi: 10.1126/science.abg3055.

39. Walker AS, Vihta K-D, Gethings O, Pritchard E, Jones J, House T, et al. Increased infections, but not viral burden, with a new SARS-CoV-2 variant. medRxiv. 2021:2021.01.13.21249721. doi: 10.1101/2021.01.13.21249721.

40. Chowell G, Hyman JM, Bettencourt LMA, Castillo-Chavez C. Mathematical and statistical estimation approaches in epidemiology: Springer, Dordrecht; 2009.

41. Graham MS, Sudre CH, May A, Antonelli M, Murray B, Varsavsky T, et al. Changes in symptomatology, reinfection, and transmissibility associated with the SARS-CoV-2 variant B.1.1.7: an ecological study. The Lancet Public health. 2021. Epub 2021/04/16. doi: 10.1016/s2468-2667(21)00055-4. PubMed PMID: 33857453.

42. Mikszewski A, Stabile L, Buonanno G, Morawska L. The airborne contagiousness of respiratory viruses: A comparative analysis and implications for mitigation. Geoscience Frontiers. 2021:101285. doi: <https://doi.org/10.1016/j.gsf.2021.101285>.

43. Campbell F, Archer B, Laurenson-Schafer H, Jinnai Y, Konings F, Batra N, et al. Increased transmissibility and global spread of SARS-CoV-2 variants of concern as at June 2021. Euro surveillance : bulletin Europeen sur les maladies transmissibles = European communicable disease bulletin. 2021;26(24). Epub 2021/06/19. doi: 10.2807/1560-7917.es.2021.26.24.2100509. PubMed PMID: 34142653; PubMed Central PMCID: PMCPMC8212592.

44. European Centre for Disease Prevention and Control. Implications for the EU/EEA on the spread of the SARSCoV-2 Delta (B.1.617.2) variant of concern Stockholm: ECDC; 2021. Available from: <https://www.ecdc.europa.eu/en/publications-data/threat-assessment-emergence-and-impact-sars-cov-2-delta-variant>.

45. Dagpunar J. Interim estimates of increased transmissibility, growth rate, and reproduction number of the Covid-19 B.1.617.2 variant of concern in the United Kingdom. medRxiv. 2021:2021.06.03.21258293. doi: 10.1101/2021.06.03.21258293.

46. Dhar MS, Marwal R, Radhakrishnan V, Ponnusamy K, Jolly B, Bhoyar RC, et al. Genomic characterization and Epidemiology of an emerging SARS-CoV-2 variant in Delhi, India. medRxiv. 2021:2021.06.02.21258076. doi: 10.1101/2021.06.02.21258076.

47. Scientific Pandemic Influenza Group on Modelling (SPIMO) Operational sub-group. Consensus statement on COVID-19 [updated 3 June 2021]. Available from: <https://www.gov.uk/government/publications/spi-m-o-consensus-statement-on-covid-19-3-june-2021>.

48. von Wintersdorff C, Dingemans J, van Alphen L, Wolffs P, Veer B, Hoebe C, et al. Infections caused by the Delta variant (B.1.617.2) of SARS-CoV-2 are associated with increased viral loads compared to infections with the Alpha variant (B.1.1.7) or non-Variants of Concern. 2021. doi: 10.21203/rs.3.rs-777577/v1.
