# Supplementary figures and images for: Analysis of two choir outbreaks acting in concert to characterize long- range transmission risks through SARS-CoV-2, Berlin, Germany, 2020
Source: PLoS One. 2022 Nov 17;17(11):e0277699. doi: 10.1371/journal.pone.0277699 (PMC9671375; doi:10.1371/journal.pone.0277699)

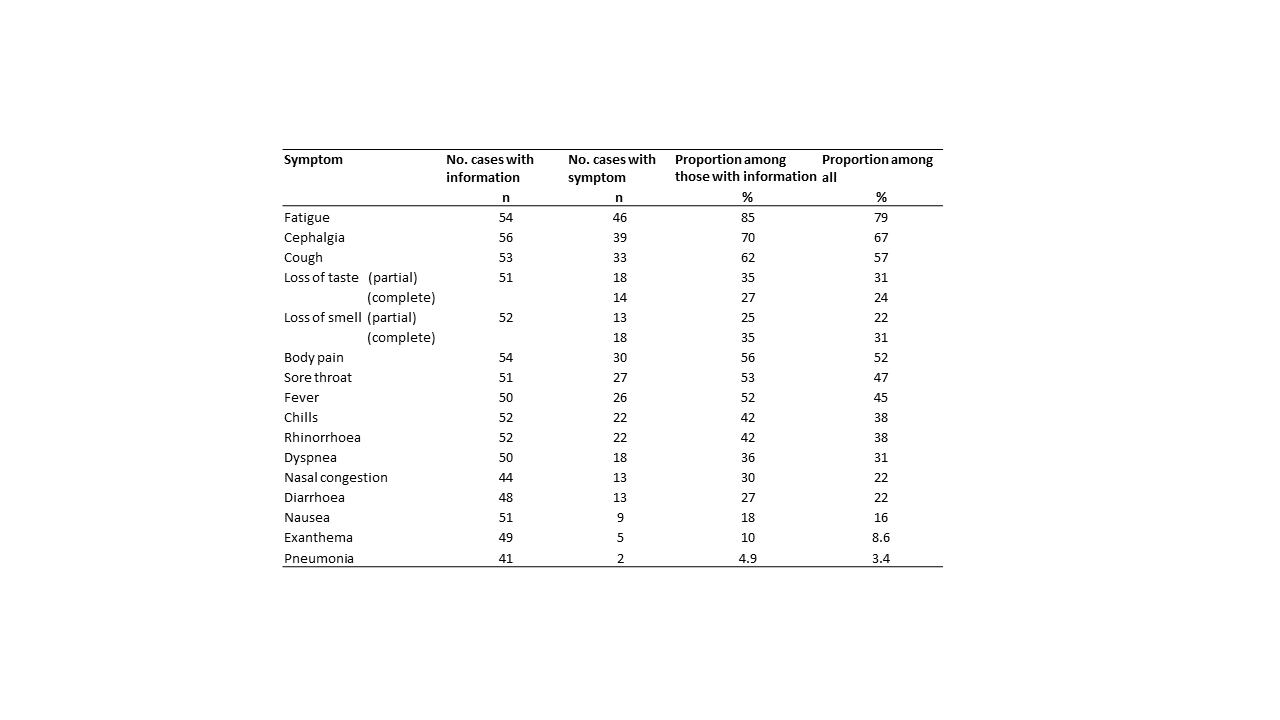

Supplement: S1 Table — (TIF) [file pone.0277699.s002.tif]

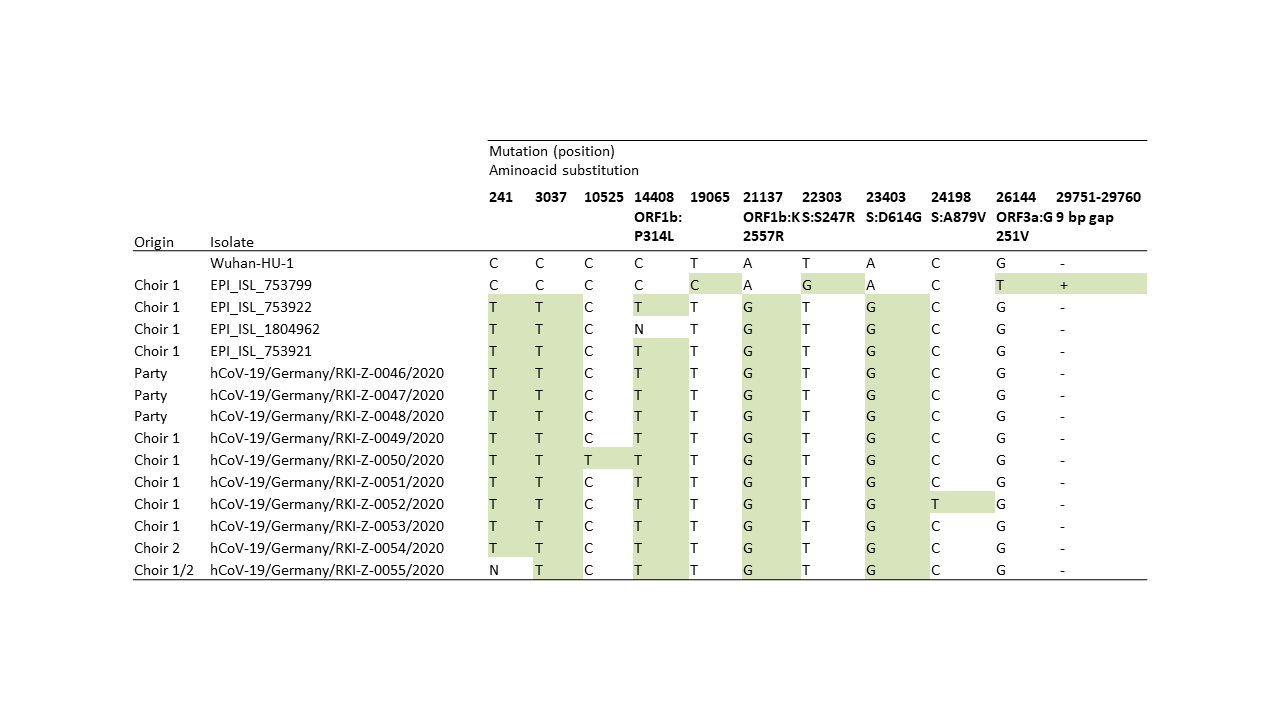

Supplement: S2 Table — (TIF) [file pone.0277699.s003.TIF]

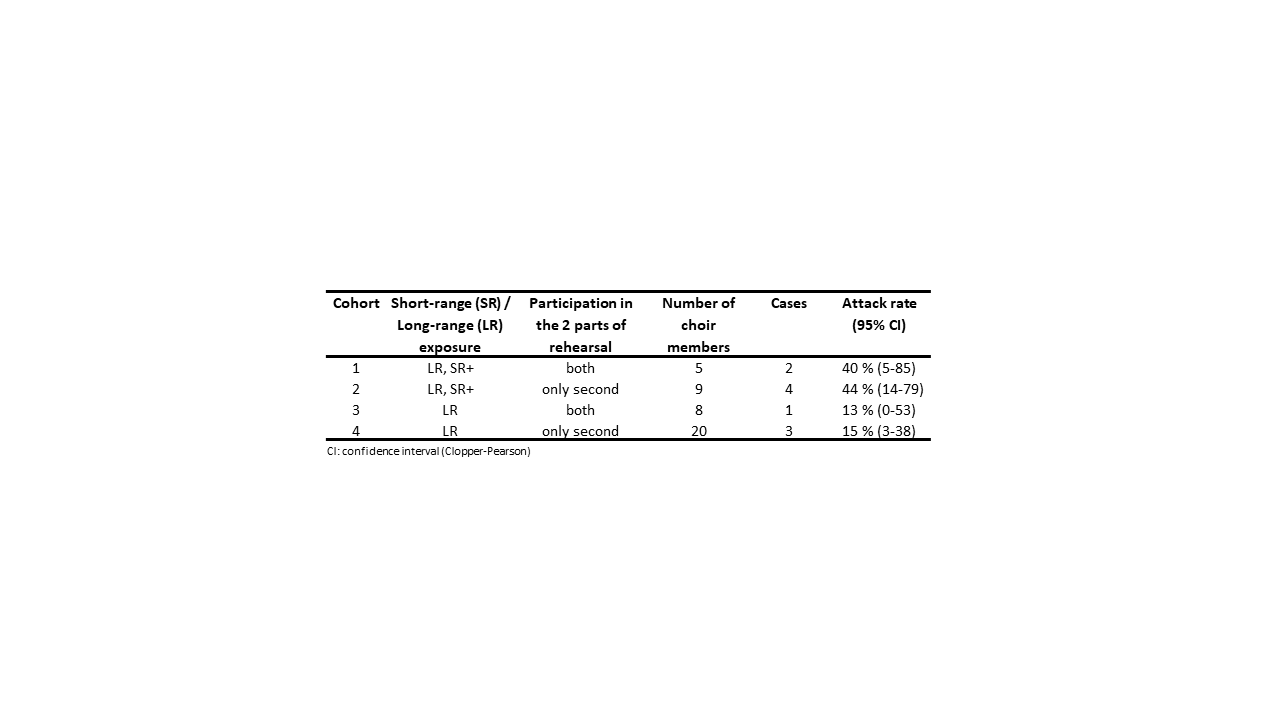

Supplement: S3 Table — (TIF) [file pone.0277699.s004.tif]

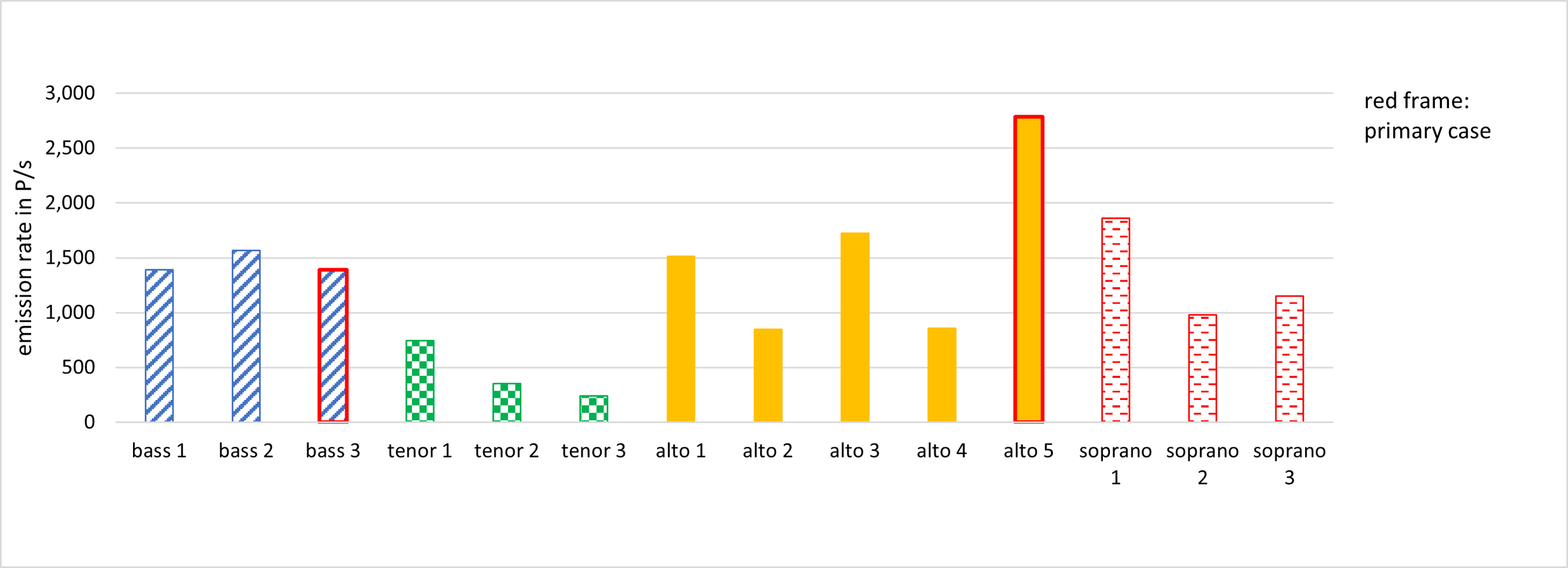

Supplement: S1 Fig — (TIF) [file pone.0277699.s005.tif]

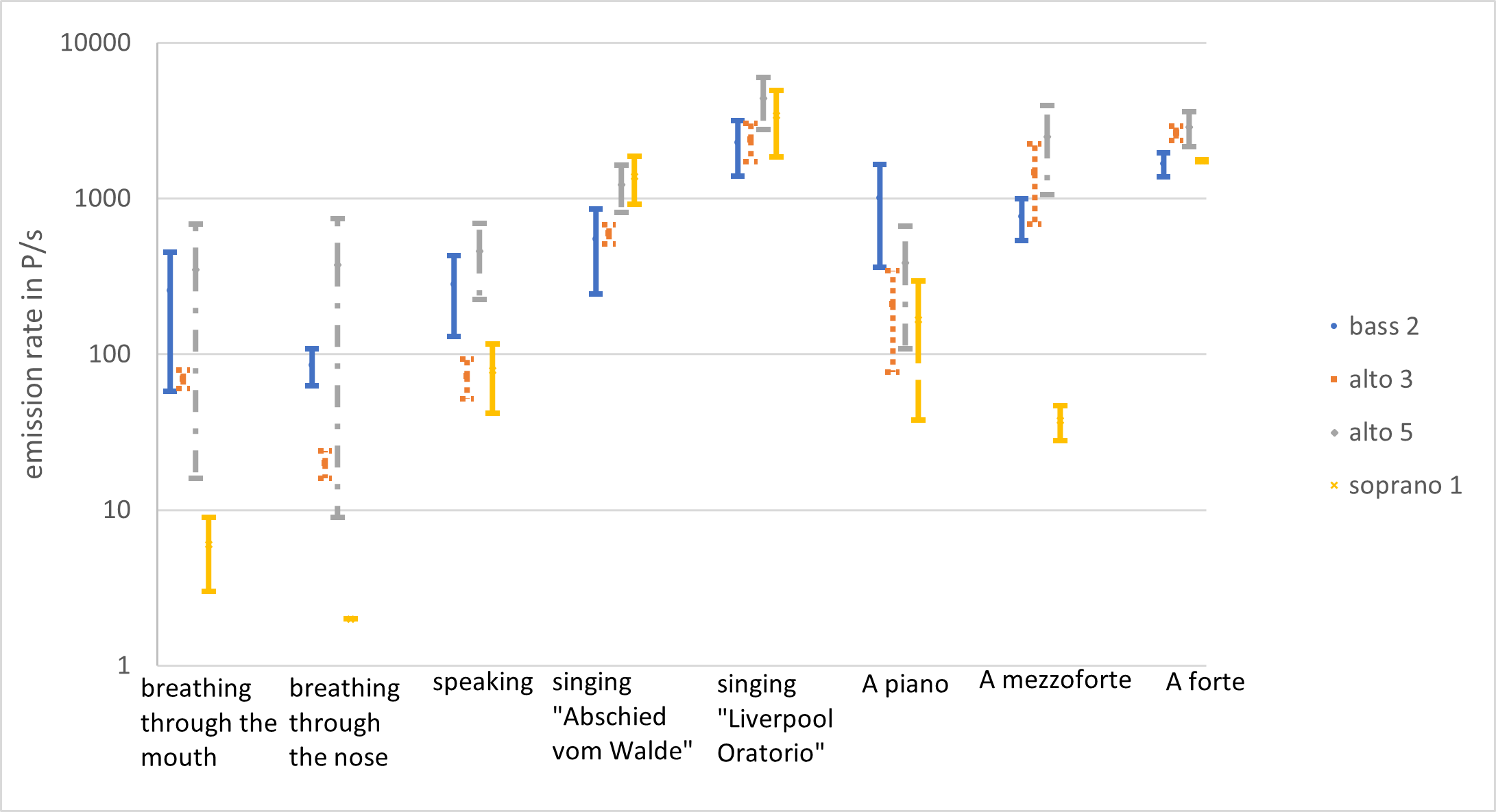

Supplement: S2 Fig — (TIF) [file pone.0277699.s006.tif]
