# Supplementary material for: Analysis of two choir outbreaks acting in concert to characterize long- range transmission risks through SARS-CoV-2, Berlin, Germany, 2020
Source: PLoS One. 2022 Nov 17;17(11):e0277699. doi: 10.1371/journal.pone.0277699 (PMC9671375; doi:10.1371/journal.pone.0277699)
Supplement: S1 File — (DOCX) [file pone.0277699.s007.docx]

| **Term** | **Description** |
| --- | --- |
| Dose | Integer number of potential pathogens, either respiratory aerosol particles potentially (or partly) carrying virions, or virions |
| Inhalation dose | Dose inhaled by a susceptible |
| Quantum | Inhalation dose sufficient to infect 63% of the susceptible |
| γ | Particle inhalation dose sufficient to infect 63% of the susceptible |
| AP_50_ | Particle inhalation dose sufficient to infect 50% of the susceptible |
| ID_50_ | Virion inhalation dose sufficient to infect 50% of the susceptible |
| Quanta dose | Dose divided by the quantum; dimensionless, fractional |
| Emission rate | Dose exhaled per unit time (hour or second) |
| Quanta emission rate | Quanta dose exhaled per unit time |
| Concentration | Dose of airborne, potential pathogens contained inside a unit volume (m³) of air |
| Viral load | Number of virions per ml of bodily fluid, determined by qRT-PCR |
| Prior distribution | Statistical distribution available before the outbreak investigation |
| Posterior distribution | Refined likelihood distribution resulting from the outbreak investigation |

S1 Glossary. Description of terms.
